# Perceived organisational support, psychological resilience and turnover intention among hospital nurses: mediation and network analyses

**DOI:** 10.1186/s12912-026-04499-x

**Published:** 2026-03-04

**Authors:** Jianzhong Ding, Jiquan Zhang, Wen Luo, Mengting Zhu, Xuan Fu, Yang He, Shunyang Liu, Jijun Wu, Xiaoli Zhong

**Affiliations:** 1 School of Basic Medical Sciences ＆ School of Nursing, Chengdu University, Chengdu, Sichuan China; 2 Department of Nursing, Deyang People’s Hospital, Deyang, Sichuan China; 3 College of Nursing, Chengdu University of Traditional Chinese Medicine, Chengdu, China

**Keywords:** Perceived organisational support, Psychological resilience, Turnover intention, Nurses, Network analysis

## Abstract

**Background:**

Perceived organisational support (POS) and psychological resilience help protect nurses from turnover intention (TI), but most studies have used only total scores, so it is unclear which concrete support or resilience elements are most influential.

**Objective:**

To test whether psychological resilience mediates the association between POS and turnover intention in hospital nurses and to identify, through network analysis, the POS and resilience items most closely linked to turnover-related cognitions.

**Methods:**

A cross-sectional survey was conducted among 439 nurses from a Grade A tertiary hospital in Sichuan Province, China. The Survey of Perceived Organisational Support (SPOS), the 10-item Connor–Davidson Resilience Scale (CD-RISC-10) and the Turnover Intention Questionnaire (TIQ) were administered. Spearman correlations and mediation modelling with 2000 bias corrected and accelerated bootstrap resamples were used to examine construct level associations, with a covariate adjusted sensitivity analysis (gender, years of work experience and job satisfaction). Item level associations were estimated using EBICglasso networks based on polychoric correlations. Expected influence (EI), bridge expected influence (bridge EI), bootstrap accuracy, case-dropping stability, and split-sample network comparison tests were performed.

**Results:**

POS was positively associated with resilience (ρ = 0.32, *p* < 0.001), and both were negatively associated with turnover intention (POS–TI: ρ = −0.33; resilience–TI: ρ = −0.36; *p* < 0.001). Mediation analysis indicated a significant indirect association between POS and lower turnover intention through resilience (standardised β_a×b = −0.104, 95% CI −0.152 to −0.058), which remained after covariate adjustment. In the three community network comprising POS, resilience and turnover intention, the item “My organisation cares about my opinions” emerged as a key bridge and was conditionally associated with “I do not get discouraged by failure.” Nodes with high expected influence included “My organisation cares about my well-being,” “I can achieve my goals even when faced with obstacles,” “The accumulation of experience has made me stronger,” and “Are you considering resigning from your current job?” Bootstrap based stability analyses suggested acceptable robustness, and split sample network comparison tests did not indicate statistically significant differences.

**Conclusion:**

Higher POS was associated with lower turnover intention, with a statistically significant indirect association through resilience at the construct level. At the item level, a small set of support and resilience contents, particularly opinion-related organisational care and persistence-oriented resilience, showed greater structural prominence and may help inform more targeted retention strategies in nursing management.

**Clinical trial number:**

Not applicable.

**Supplementary Information:**

The online version contains supplementary material available at 10.1186/s12912-026-04499-x.

## Introduction

Global health systems continue to face a persistent shortage of nurses, with deterioration in some regions [1]. International reports indicate that several million additional health workers will be needed in the coming decade, and nurses account for the largest single group within that gap [2]. Population ageing, the growing burden of chronic disease, and increased service demands after the COVID-19 pandemic all contribute to this deficit [3]. When existing nursing staff leave or intend to leave, the shortage becomes more visible at the bedside. Units operate with fewer staff, the workload for those who remain increases, and the risk of adverse events and patient dissatisfaction rises [4]. Preventing turnover at an early stage is therefore a pragmatic and cost effective strategy for hospitals that cannot immediately expand recruitment [5, 6].

Turnover intention is widely recognised as the most proximal and reliable attitudinal predictor of actual turnover [7]. Resignations are rarely abrupt in nursing. They first form an intention to quit, to look for another post, or to work elsewhere. If the determinants of this intention can be identified and modified, organisations may be able to retain experienced staff and stabilise teams [8]. Studies in nursing have also shown that turnover intention cannot be explained by salary or workload alone. Perceptions of fairness, professional respect, psychological safety, career development, and support from the organisation all play important roles [9–11]. These findings suggest that organisational and individual resources should be considered together.

Perceived organisational support (POS) is an important organisational resource in this context [12]. POS refers to the extent to which employees believe that the organisation values their contribution and cares about their well-being [13]. Nursing studies have reported that higher POS is linked to higher job satisfaction, stronger organisational commitment, lower burnout, and lower turnover intention [14–16]. In other words, nurses who feel supported are less likely to consider leaving their organisation. These patterns align with social exchange theory, which proposes that perceived organisational care encourages reciprocal attitudes and a stronger intention to remain [17–19]. A supportive climate also communicates that the organisation will invest in employees, which reduces uncertainty and the need to seek alternative employment [20]. Across settings such as acute wards, primary care and long term care, similar patterns have been reported, particularly where organisations listen to nurses’ views and involve them in decisions. This suggests that POS may operate as a protective factor across contexts [21, 22].

Psychological resilience represents the individual side of this resource system. Resilience is the capacity to adapt successfully when facing stress, failure, or adversity [23]. Nurses, especially those working in tertiary hospitals, often encounter high patient acuity, rapid patient turnover, and emotionally demanding communication with families [24, 25]. Nurses with higher resilience report better work engagement and lower levels of depression, compassion fatigue, and turnover intention [26]. A growing number of studies also show that resilience can be strengthened through training, supervision, and supportive leadership [27–29]. This means that resilience is not only associated with turnover intention but may also be a feasible target for intervention.

Theoretical perspectives suggest that POS and resilience may be linked. Social exchange theory indicates that supportive organisational practices can foster trust, reciprocity, and positive work attitudes [17–19]. Conservation-of-resources theory is also consistent with the view that contextual resources are associated with the development and protection of personal resources under stress [30]. Consistent with these perspectives, prior studies have reported that workplace support is associated with stronger personal resources (resilience) and more favourable work-related outcomes [31]. In nursing settings, POS has also been associated with greater confidence, persistence, and sense of control [32, 33].

However, several gaps remain in the current literature. First, most studies have relied on total scores of POS, resilience, and turnover intention [34, 35]. Although total scores are informative, they may mask important heterogeneity within constructs [36]. For example, POS items reflecting voice and participation may relate differently to turnover intention than items reflecting general organisational concern [37]; similarly, resilience items on perseverance or goal pursuit may differ from items on humour or emotion regulation [38]. Second, only a few nursing studies have applied network analysis to organisational and psychological variables. Network analysis models items as nodes and conditional associations as edges, allowing influential items and bridge items connecting communities to be identified [39]. It enables estimation of network accuracy and stability and supports robustness checks [40]. These procedures are increasingly used in psychological network research but have rarely been applied to examine how organisational support and resilience relate to nurses’ turnover intention.

In view of these gaps, we designed this study with two aims. The first aim was to examine, at the construct level, whether resilience accounted for part of the POS–turnover intention association among hospital nurses. The second aim was to map, at the item level, the interrelations among POS, resilience, and turnover intention, identify items with high expected influence and bridge expected influence, and evaluate network accuracy and stability. By combining a traditional mediation model with a modern network approach, this study moves from asking “Does support matter?” to asking “Which specific aspects of support and which aspects of resilience matter most for keeping nurses in their jobs?” Given the cross-sectional design, mediation results were interpreted as statistical evidence of indirect association rather than temporal or causal effects.

## Methods

### Participants

We conducted a cross-sectional survey using convenience sampling among clinical nurses at a Grade A tertiary hospital in Sichuan Province, China. A total of 448 nurses were approached and invited to participate. During data screening, 9 questionnaires were excluded due to excessively short completion times and/or duplicate response patterns, yielding a final analytic sample of 439 nurses, corresponding to 97.99% valid responses. Eligible participants were (a) registered nurses holding a valid Chinese nursing practice licence, (b) currently employed in the hospital, and (c) with at least one year of clinical experience at the current hospital. Exclusion criteria were as follows: (a) nurses who were on leave (including sick or maternity leave); (b) nurses who were in the process of resigning; and (c) nurses in rotation during the survey period. After eligibility was confirmed, participants provided informed consent prior to completing the questionnaire. The study protocol was approved by the Ethics Committee of Deyang People’s Hospital (Approval No. 2024–04-016-K01).

### Measures

A structured questionnaire was administered to assess POS, psychological resilience, and turnover intention.

POS was measured with the 8-item short form of the Survey of Perceived Organisational Support [13]. The Chinese version has shown acceptable internal consistency in prior validation work (Cronbach’s α = 0.830) [41]. Items are scored on a 5-point Likert scale (1 = strongly disagree, 5 = strongly agree); items 2, 5 and 8 are reverse coded. Total scores are obtained by summing the items, with higher scores indicating stronger POS. In the present sample, Cronbach’s α was 0.804.

Psychological resilience was measured with the 10-item Connor–Davidson Resilience Scale (CD-RISC-10) [42]. The Chinese version has demonstrated high internal consistency in prior validation work (Cronbach’s α = 0.961) [43]. Items are scored on a 5-point Likert scale (0 = almost never, 4 = almost always). Total scores are obtained by summing the items, with higher scores reflecting greater resilience. In the present sample, Cronbach’s α was 0.942.

Turnover intention was assessed using the Turnover Intention Questionnaire (TIQ) adapted from Spector [44]. A Chinese version has been translated, culturally adapted, and validated for use among nurses (Cronbach’s α = 0.770) [45]. Items are rated on a 4-point Likert scale (1 = strongly disagree, 4 = strongly agree). Total scores are obtained by summing the items, with higher scores indicating stronger turnover intention. In the present sample, Cronbach’s α was 0.863.

### Data analysis

All analyses were conducted in R (version 4.5.1). Descriptive statistics were calculated for demographic variables and for the POS, resilience, and turnover intention total scores. Categorical variables were presented as frequencies and percentages, and continuous variables as mean (SD) and median (IQR), as appropriate. Normality of the three scale totals was assessed using Shapiro–Wilk tests and visual diagnostics (Supplementary Fig. S1). Given deviations from normality of item responses, non-parametric analyses (Spearman correlations) were used for construct-level associations (Supplementary Table 1, Supplementary Table 2). All scale totals were calculated by summing their respective items.

To maximise reproducibility, we prespecified the data handling and estimation details for the mediation model. Scale scores for perceived organisational support (POS), resilience (CD-RISC-10), and turnover intention (TI) were computed as summed item scores. In the main model, these summed scores were treated as continuous observed variables, and standardised coefficients (β) are reported. The model was estimated in lavaan using maximum likelihood with bias corrected and accelerated (BCa) bootstrap confidence intervals based on 2000 resamples. We report total (c), direct (c′), and indirect (a×b) effects [46]. Because this simple mediation model is just-identified (df = 0), global fit indices (CFI, TLI, RMSEA, SRMR) are mathematically saturated and therefore not informative for model evaluation; they are provided for completeness only. As a robustness check, we re-estimated the model with key covariates (gender, years of work experience, and job satisfaction). Results of the adjusted model were consistent with the primary pattern (Supplementary Table 3). Therefore, inference is based on convergence between the primary and covariate-adjusted specifications.

To move beyond scale-level associations and explore item-level interactions, we estimated partial correlation networks in which each item from the POS, CD-RISC-10 and TIQ scales was treated as a node and edges represented conditional associations between items. Network estimation was conducted using the EBICglasso procedure (graphical LASSO with the extended Bayesian information criterion) implemented in the qgraph package [36]. Because all items were ordinal, we first obtained a polychoric correlation matrix with the cor_auto() function in qgraph, and this matrix was then used as the input for EBICglasso. After inspecting alternative tuning values, the regularisation parameter was set to γ = 0.10 so that the resulting networks were sparse and substantively interpretable [40]. For node importance we focused on one-step expected influence (EI), which takes into account both the sign and the magnitude of adjacent edges; strength, closeness and betweenness were also calculated, but were interpreted only if they reached acceptable stability in bootstrapping [47].

To identify items that connect the three predefined communities (POS, resilience, turnover intention), we computed bridge-centrality indices (one-step bridge expected influence and bridge strength) in R using the networktools package, which provides dedicated functions for detecting bridging nodes in psychological networks [48]. For all node-level and bridge-level indices we obtained non-parametric bootstrap confidence intervals and case-dropping correlation-stability coefficients to evaluate precision and robustness. Finally, to examine the reproducibility of the estimated networks, the full sample was randomly divided into two subsamples of approximately equal size (*n* ≈ 220), and the two networks were compared with the NetworkComparisonTest package for both network invariance and global-strength invariance. *p*-values were adjusted using the Benjamini–Hochberg procedure [49].

## Results

### Sample characteristics

A total of 439 clinical nurses were included in the analysis. Most participants were female (93.4%), 30–40 years old (58.5%), married (67.0%), and held a bachelor’s degree (90.0%); more than half had more than 10 years of work experience (54.0%). Nearly half reported more than five night shifts per month, and most rated their sleep as fair or good. The mean total scores were 32.71 (5.41) for perceived organisational support (POS), 30.92 (6.21) for psychological resilience (CD-RISC-10), and 9.89 (3.59) for turnover intention (TI). Full descriptive statistics for demographic and study variables are shown in Table 1.

**Table 1:** Descriptive statistics of participants

Variable	Total (N = 439)
Gender, n (%)	
Female	410 (93.4%)
Male	29 (6.6%)
Age, n (%)	
<30	109 (24.8%)
30–40	257 (58.5%)
>40	73 (16.6%)
Marital Status, n (%)	
Single	93 (21.2%)
Married	294 (67%)
Divorced	41 (9.3%)
Widowed	11 (2.5%)
Degree, n (%)	
High school	33 (7.5%)
Bachelor	395 (90%)
Postgraduate	11 (2.5%)
Years of work experience, n (%)	
≤2	26 (5.9%)
3–5	57 (13%)
5–10	119 (27.1%)
>10	237 (54%)
Position, n (%)	
Chief head nurse	3 (0.7%)
Charge nurse	26 (5.9%)
Supervisor nurse	59 (13.4%)
Staff nurse	351 (80%)
Professional title, n (%)	
Nurse	51 (11.6%)
Senior nurse	196 (44.6%)
Supervisor nurse	168 (38.3%)
Chief nurse	24 (5.5%)
Number of night shifts, n (%)	
0	84 (19.1%)
1–2	67 (15.3%)
3–5	71 (16.2%)
>5	217 (49.4%)
Anxiety, n (%)	
No	380 (86.6%)
Yes	59 (13.4%)
Medication use, n (%)	
No	389 (88.6%)
Yes	50 (11.4%)
Chronic illness, n (%)	
No	239 (54.4%)
Yes	200 (45.6%)
Sleep quality, n (%)	
Very poor	5 (1.1%)
Poor	73 (16.6%)
Fair	203 (46.3%)
Good	111 (25.3%)
Very good	47 (10.7%)
Job satisfaction, n (%)	
Dissatisfied	54 (12.3%)
Neutral	279 (63.6%)
Satisfied	106 (24.1%)
POS, mean ± SD	32.71 ± 5.41
CD-RISC-10, mean ± SD	30.92 ± 6.21
TI, mean ± SD	9.89 ± 3.59

Position refers to job post (e.g., staff nurse, charge nurse), whereas professional title refers to rank (e.g., nurse, senior nurse, supervisor nurse, chief nurse). Medication use indicates current use of prescribed medication (yes/no). POS, Perceived Organisational Support; CD-RISC-10, 10-item Connor-Davidson resilience scale; TI, Turnover intention

### Mediation model

**Fig. 1:**
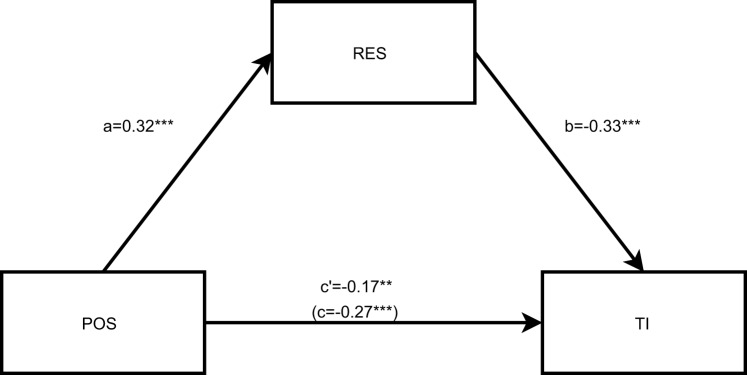
Mediation model between POS, RES and TI as unitary constructs. POS is the independent variable, RES the mediating variable and TI the dependent variable. Total effects (**c**) and direct effects (c’) are also illustrated in the model. Bootstrapped standardised β_a = 0.317, 95% CI [0.225, 0.409], standardised β_b = −0.331, 95% CI [−0.437, −0.227], total effect standardised β_c = −0.274, 95% CI [−0.357, −0.193], direct effect standardised β_c’ = −0.170, 95% CI [−0.262, −0.078], indirect effect standardised β_a×b = −0.104, 95% CI [−0.152, −0.058]. POS, perceived organisational Support; RES, resilience; TI, turnover intention; CI, confidence interval. * *p* < 0.05, ** *p* < 0.01, *** *p* < 0.001

Spearman correlations showed that POS was positively associated with resilience and that both variables were negatively associated with turnover intention (Supplementary Table 2). Specifically, POS was moderately and positively correlated with resilience (ρ = 0.32, *p* < 0.001), POS was moderately and negatively correlated with TI (ρ = −0.33, *p* < 0.001), and resilience was moderately and negatively correlated with TI (ρ = −0.36, *p* < 0.001). On this basis, a mediation model was estimated to test whether resilience accounted for part of the POS–TI association (Fig. 1; Supplementary Table 3). In the baseline model, POS had a negative total association with TI (standardised β_c = −0.274, 95% CI [−0.357, −0.193]) and was positively associated with resilience (standardised β_a = 0.317, 95% CI [0.225, 0.409]); resilience was negatively associated with TI (standardised β_b = −0.331, 95% CI [−0.437, −0.227]). The bootstrapped indirect effect was significant (standardised β_a×b = −0.104, 95% CI [−0.152, −0.058]) (Supplementary Table 3). After adjusting for gender, years of work experience, and job satisfaction, the indirect effect remained significant, whereas the direct path from POS to TI was attenuated and no longer statistically significant (Supplementary Table 3). Given the cross-sectional design, these findings should be interpreted as evidence consistent with the hypothesised pattern of association, rather than as temporal or causal effects.

### Network accuracy and stability

Before interpreting specific nodes, we first evaluated whether both estimated networks were accurate and robust. For the two-community POS–TI network and the three-community POS–RES–TI network, non-parametric bootstrapping produced generally narrow 95% confidence intervals for most edge weights (Supplementary Fig. S2a, c), indicating that the partial correlations were estimated with acceptable precision. Case-dropping bootstraps further showed that the main node indices and edge weights remained stable when progressively larger portions of the sample were removed (Supplementary Fig. S3). In the POS–TI network, expected influence (EI), strength and edge weights all reached the recommended level of stability (CS = 0.672 for all three indices), indicating that more than half of the cases could be dropped while preserving correlations ≥ 0.70 with the original estimates. In the POS–RES–TI network, stability was even higher for EI (CS = 0.749) and remained acceptable for edge weights (CS = 0.749), while strength showed a slightly lower but still usable value (CS = 0.595). The largely overlapping confidence bands for EI and strength further supported our decision to prioritise EI in subsequent interpretations. In contrast, distance-based indices such as closeness and betweenness displayed clearly lower CS values in both networks and were therefore not used as primary indicators. Given this difference in stability, subsequent interpretations focused on the three-community POS–RES–TI network.

**Fig. 2:**
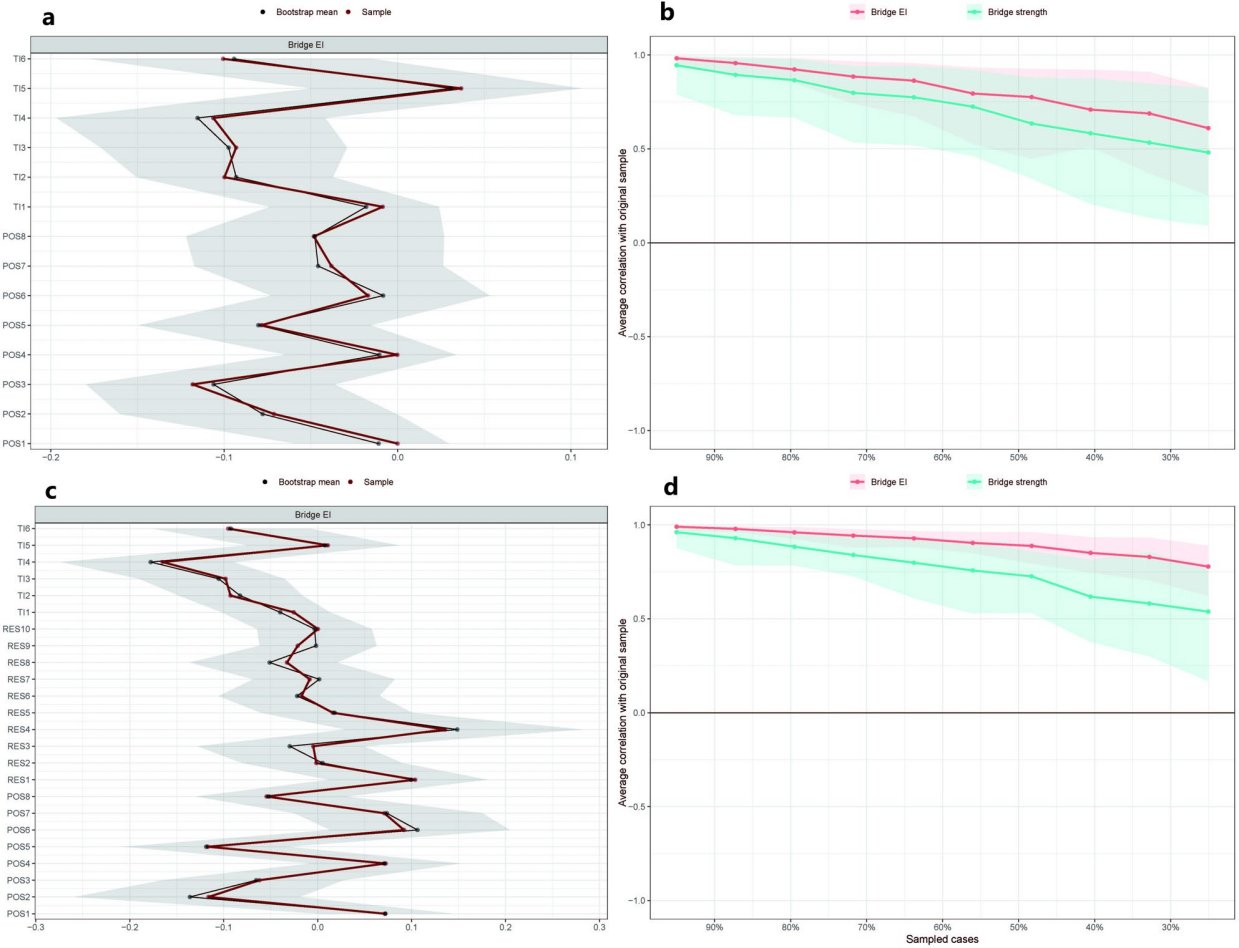
Accuracy and stability of bridge centrality indices using a nonparametric and case-dropping bootstrap approach. The nonparametric bootstrap method estimates the accuracy of the bridge EI. Red dots represent each node’s corresponding bridge EI, from the highest to the lowest. Gray shading represents the 95% CI for the bridge EI. A narrower gray shadow means better accuracy for the bridge EI. The case-dropping bootstrap method increasingly dropped cases from the original sample and compared the centrality indices in the new versus the original dataset. The x-axis indicates the proportion of the dataset that can be dropped. The y-axis represents the correlation between the original bridge centrality indices and those after the cases were dropped with a 95% probability. The shades represent the 95% CI for the bridge centrality indices. a, accuracy of the bridge EI in the POS-TI network. b, stability of bridge strength and bridge EI in the POS-TI network. c, accuracy of the bridge EI in the POS-RES-TI network. d, stability of bridge strength and bridge EI in the POS-RES-TI network. EI, expected influence; POS, perceived organisational Support; RES, resilience; TI, turnover intention

Bridge-centrality indices were evaluated in parallel. As shown in Fig. 2a and c (Supplementary Fig. S2b, d), bootstrap confidence intervals were relatively narrow for bridge expected influence but wider for bridge strength, suggesting that bridge EI was estimated more precisely. Case-dropping results in Fig. 2b and d indicated that, in the POS–RES–TI network, bridge EI reached an acceptable level of stability (CS = 0.672), whereas bridge strength was less stable (CS = 0.282). In the POS–TI network, both bridge indices were more sensitive to case deletion (CS_bridgeEI = 0.282; CS_bridgeStrength = 0.128), so these values should be treated as exploratory. Because EI and bridge EI showed the most favourable combination of precision and stability, and are increasingly recommended for signed psychological networks, EI was used as the primary centrality indicator in the subsequent presentation.

### Bridge and node centrality

**Fig. 3:**
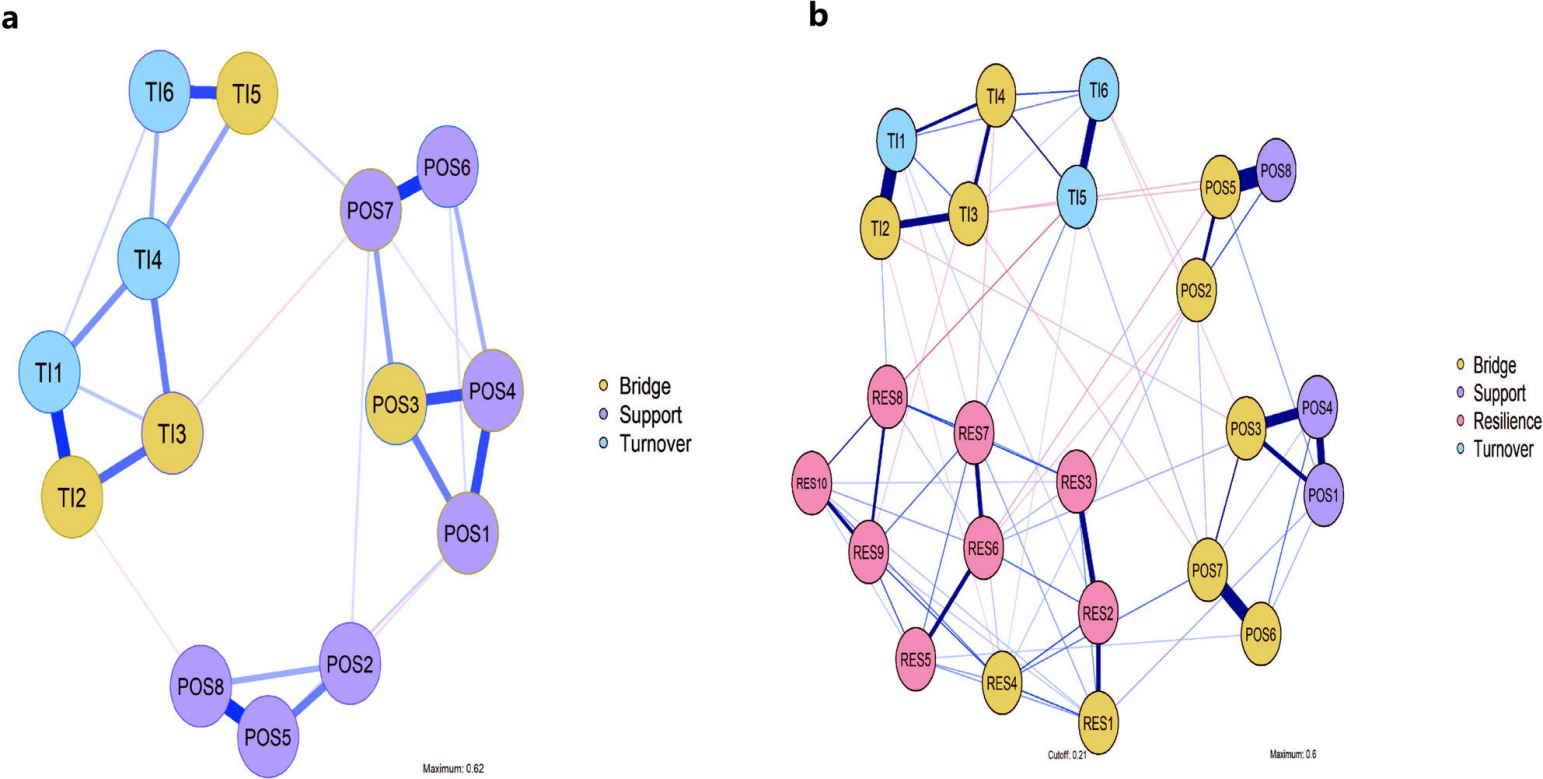
Estimated network structures. a, b, network structures of POS and TI (**a**) and POS, RES and TI (**b**) in 439 nurses. Each node represents a specific item, and nodes from the same questionnaire are grouped and color-coded accordingly. Blue nodes represent individual items from the turnover intention questionnaire (TIQ). Purple nodes represent individual items from the survey of perceived organisational support (SPOS). Pink nodes represent individual items from the 10-item Connor–Davidson resilience scale (CD-RISC-10). The thickness and length of the edges reflect the strength of the partial correlations between nodes; thicker or shorter edges indicate stronger associations. Edge colors represent the direction of partial correlations, with blue indicating positive and red indicating negative relationships. Bridge was defined as nodes showing high values on both bridge strength and bridge EI, and are highlighted in yellow. POS, perceived organisational Support; RES, resilience; TI, turnover intention

The two estimated networks are shown in Fig. 3. In the POS–TI network (Fig. 3a), the node with the highest bridge expected influence was TI5 (“Given your current situation and qualifications, how likely do you think it is to find a suitable position in another organisation”). TI5 showed its strongest cross-community edge with POS7 (“My organisation cares about my opinions” partial *r* = 0.095), indicating that nurses who feel that their views are taken seriously are less likely to centre thoughts about easily finding alternative employment. However, because bridge indices in this two-community model were only modestly stable (CS_bridgeEI = 0.282, CS_bridgeStrength = 0.128), these cross-cluster links should be interpreted with caution and mainly as signals of potential transmission routes rather than definitive intervention targets.

**Fig. 4:**
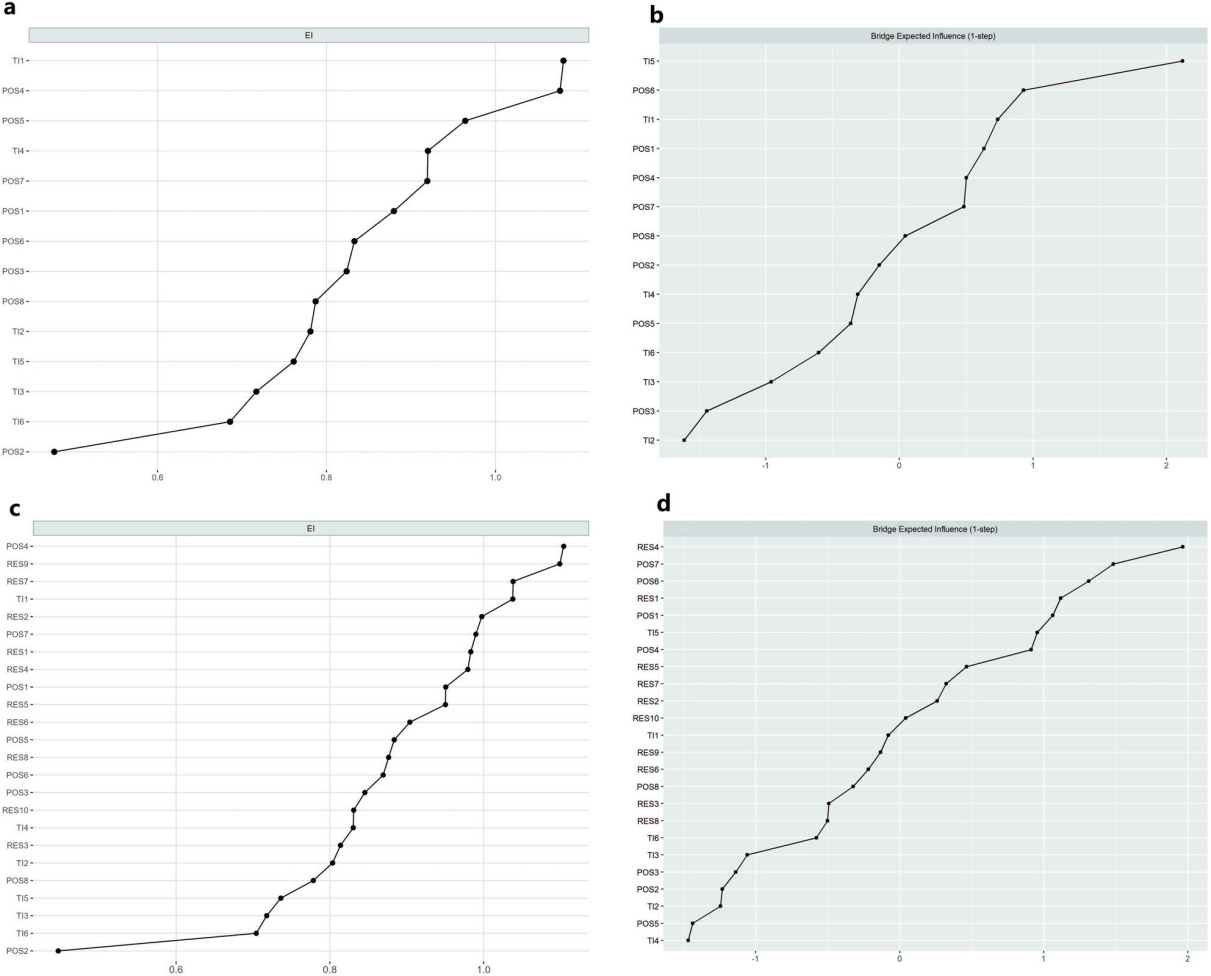
Node and bridge centrality indices. The EI (1-step) and bridge EI (1-step) were used in the present study. 1-step EI is defined as the sum of all the edges extending from a node, including both negative and positive ones. 1-step bridge EI is the sum of edge weights extending from one node to all nodes in other communities. a, b, EI (**a**) and bridge EI (**b**) of each node, from the highest to the lowest, of the POS–TI network. c, d, EI (**c**) and bridge EI (**d**) of each node, from the highest to the lowest, of the POS–RES-TI network. POS, perceived organisational Support; RES, resilience; TI, turnover intention

In the POS–RES–TI network (Fig. 3b), POS7 emerged as the key bridging node. POS7 was positively and conditionally connected with RES4 (“I do not get discouraged by failure” partial *r* = 0.124), suggesting that organisational practices that listen to and respect nurses’ opinions may be associated with improvements in a “not easily discouraged” resilience profile, which in turn is linked to lower turnover-related cognitions. The complete edge-weight matrices corresponding to Fig. 3a and b are provided in Supplementary Tables S4 and S5. Bootstrapped difference tests for bridge EI are shown in Fig. 4b and d and in Supplementary Fig. S6.

Node-level EI plots (Fig. 4a, c), together with the bootstrapped difference tests (Supplementary Fig. S4b, d), revealed a consistent centrality pattern. In the POS–TI network, two items were clearly more central than the rest: TI1 (“Are you considering resigning from your current job”) and POS4 (“My organisation cares about my well-being”). In the POS–RES–TI network, four nodes formed the top EI tier: POS4, RES9 (“I can achieve my goals even when faced with obstacles”), RES7 (“The accumulation of experience has made me stronger”) and TI1. Together, these results indicate that the structurally most embedded elements of the joint network are two supportive-climate items (care for well-being and care for opinions), two perseverance-type resilience items, and the core turnover-intention item. Additional centrality indices (strength, closeness, betweenness) and bridge strength rankings are presented in Supplementary Figs. S7–S8 to facilitate comparison across metrics.

### Network comparison tests

Finally, we examined whether the network structure was reproducible in subgroups of the same sample. The original dataset was randomly split into two subsamples of similar size, and the Network Comparison Test found no statistically significant differences in either network structure (network invariance: *M* = 0.329, *p* = 0.099) or overall connectivity (global strength: S = 0.669, *p* = 0.218). Visual inspection of the two networks (Supplementary Fig. S5a, b) yielded very similar layouts and bridge patterns, and the corresponding bridge-EI rankings for the two subsamples (Supplementary Fig. S5c, d) showed similar high-ranking items, which further supports the reproducibility of the identified bridges. These findings support the internal robustness of the estimated networks and indicate that the key bridging items identified above are not artefacts of a particular split.

## Discussion

This study examined the association between POS, psychological resilience, and turnover intention among hospital nurses using a combined construct-level mediation analysis and item-level network approach. In the present sample, higher POS was associated with lower turnover intention, and this association was statistically consistent with an indirect pathway through resilience; at the item level, “the organisation cares about my opinions,” “the organisation cares about my well-being,” “not getting discouraged by failure,” “becoming stronger through experience,” and “considering resignation” were among the most structurally influential elements in the joint network. These findings extend prior turnover research that has mainly relied on total scores by identifying specific support- and resilience-related components that may be more actionable for workforce retention planning. Importantly, because the design is cross-sectional, the mediation results should be interpreted as pattern-consistent indirect associations rather than evidence of temporal or causal mechanisms.

At the construct level, our findings are broadly aligned with prior nursing evidence showing that stronger organisational support is associated with lower turnover intention and better psychosocial outcomes. A recent meta-analysis reported a robust inverse association between organisational support and nurses’ turnover intention across settings [18], and more recent studies in Chinese and international nursing samples similarly show that supportive organisational climates are associated with better engagement, lower burnout, and reduced intention to leave, although effect sizes vary across settings and workforce composition [20, 21]. Our result that resilience is negatively associated with turnover intention is also consistent with studies showing that resilience functions as a protective personal resource in high-demand clinical work environments [32]. Moreover, the indirect association pattern observed here (POS to resilience to lower turnover intention) is in line with studies modelling resource-based mechanisms in nurses, including work engagement and professional identity pathways [33]. The conceptual coherence of these findings is compatible with social exchange theory and conservation-of-resources theory: supportive institutions provide contextual resources that are reciprocated through more positive work attitudes and may help preserve personal resources under stress [17–19]. At the same time, our manuscript deliberately avoids causal phrasing and treats this framework as an explanatory lens for observed covariation, which directly addresses concerns about over-interpretation in cross-sectional mediation.

Where this study adds beyond most prior reports is at the item-network level. Earlier nurse studies often established that “support matters” or “resilience matters” at the scale level, but they rarely identified which concrete item contents anchor cross-domain connections [32]. In our three-community network, the item reflecting organisational attention to nurses’ opinions emerged as a key bridge, with meaningful conditional linkage to the resilience item on not being discouraged by failure. This pattern suggests that voice-related support may not only is associated with more favourable perceptions of the organisation but may also be linked to a psychologically specific resilience process (persistence under setbacks), which is statistically linked to turnover cognitions in this cross-sectional dataset [23]. This interpretation is plausible in light of nursing management literature showing that participatory climate, decisional involvement, and perceived fairness are tied to stronger commitment and retention intentions [22, 50]. It is also consistent with emerging psychological network work indicating that bridge nodes can represent transmission channels across symptom or construct communities, and that bridge expected influence may be more informative than unsigned metrics when positive and negative edges coexist [39]. Notably, our centrality profile highlighted both an “organisational care” item and a “resignation contemplation” item, implying that retention dynamics may involve simultaneous pressure from institutional experience and proximal quitting cognition rather than a single dominant driver.

Some differences from previous studies are also worth noting. First, several earlier papers positioned resilience more as a broad moderator or distal trait, whereas our network results suggest that specific resilience facets (persistence and goal pursuit) may be more tightly linked to turnover-related cognition than other facets such as humour based coping [51–53]. This discrepancy may reflect differences in measurement granularity (total scores and item-level modelling), occupational context (single tertiary hospital with substantial night-shift burden), and analytic strategy (partial-correlation network with regularisation and conventional regression). Second, compared with studies where direct POS to turnover paths remain significant after adjustment, our adjusted model showed attenuation of the direct path while the indirect path remained significant; this may indicate that, in this sample, resilience captures a larger share of POS-related variance once demographic and job-related covariates are considered. Such between-study variation is expected because effect decomposition is sensitive to covariate sets, local organisational climate, and scale distributions [54]. Therefore, rather than claiming superiority, we interpret our findings as complementary evidence that refines “where the association may run” under one concrete clinical context.

These results have practical implications for nurse retention policy and unit-level management. The identified high-centrality/bridge contents point to interventions that are specific, feasible, and evaluable: strengthening routines that visibly acknowledge nurses’ opinions, ensuring timely managerial response to complaints, and embedding resilience-building components focused on persistence after setbacks and goal-directed coping. Evidence from intervention literature suggests that resilience oriented programmes and supportive supervisory practices may be linked to better work-related psychological outcomes among healthcare workers [55, 56]. In practice, hospitals could combine (1) participatory governance micro-practices, with (2) brief resilience skill modules integrated into continuing education, and (3) targeted monitoring of “active resignation contemplation” as an early warning cognition. Because our strongest bridges were not uniformly distributed across all items, a focused “few-key-item” strategy may yield higher implementation efficiency than undifferentiated broad programmes.

This study has limitations. First, the single-site cross-sectional design limits temporal inference and external generalisability; longitudinal or multi-centre designs are needed to test whether the observed indirect pattern is stable over time and across organisational cultures. Second, self-reported measures may introduce common-method variance and social desirability bias, although using validated Chinese versions and reporting internal consistency in both prior and present samples improves measurement transparency. Third, although centrality and bridge indices showed acceptable robustness in the three-community model, some bridge metrics in the two-community model were less stable; therefore, we treated those links as exploratory and prioritised the better-performing indices, which is consistent with current network methodology recommendations. Fourth, turnover intention is a proximal attitudinal predictor rather than actual turnover; future work should link these network-derived targets to hard outcomes such as 6–12 months resignation records. Future studies should therefore adopt prospective designs, include objective retention endpoints, and test whether interventions targeting identified bridge contents produce measurable reductions in actual turnover.

## Conclusion

This study contributes by integrating theory-consistent construct-level modelling with item-level network evidence to clarify how organisational and personal resources are jointly associated with nurses’ turnover intention. The main message is not that one global score dominates, but that a small set of concrete perceptions and resilience responses may carry greater structural prominence. This evidence can inform hypothesis-driven intervention trials and implementation studies aimed at precision retention management in hospital nursing workforces.

## Electronic supplementary material

Below is the link to the electronic supplementary material.

Supplementary material 1

## Data Availability

The datasets generated and/or analysed during the current study are not publicly available due to ethical restrictions. However, they are available from the corresponding author upon reasonable request.

## Abbreviations

POS: Perceived organisational support
TI: Turnover intention
SPOS: Survey of Perceived Organisational Support
CD-RISC-10: 10-item Connor–Davidson Resilience Scale
TIQ: Turnover Intention Questionnaire
SD: Standard deviation
CI: Confidence interval
EBIC: Extended Bayesian information criterion
LASSO: Least absolute shrinkage and selection operator
EI: Expected influence
CS: Correlation-stability
RES: Resilience
