# Supplementary material for: Perceived organisational support, psychological resilience and turnover intention among hospital nurses: mediation and network analyses
Source: BMC Nurs. 2026 Mar 4;25:342. doi: 10.1186/s12912-026-04499-x (PMC13067519; doi:10.1186/s12912-026-04499-x)
Supplement: Supplementary file 1 — Supplementary material 1 [file 12912_2026_4499_MOESM1_ESM.docx]

**Supplementary Materials**

Supplementary Table 1. Descriptive statistics and distribution characteristics of the composite scores.

Supplementary Table 2. Spearman correlations among key study variables.

Supplementary Table 3. Standardized path coefficients and indirect effects for the mediation models.

Supplementary Table 4. Edge weight matrix of the POS-TI network.

Supplementary Table 5. Edge weights matrix of the POS-RES-TI network

Supplementary Fig. 1: Histograms and Q–Q plots

Supplementary Fig. 2: Accuracy of edge weights and bridge strength.

Supplementary Fig. 3: Stability of node centrality indices and edges.

Supplementary Fig. 4: Bootstrapped difference test of edge weights and expected influence.

Supplementary Fig. 5: Network Comparison tests of the POS-RES-TI networks of the two equally split subsamples.

Supplementary Fig. 6: Bootstrapped difference tests of bridge expected influence.

Supplementary Fig. 7: Node centrality indices: strength, betweenness, closeness, and expected influence.

Supplementary Fig. 8: Bridge strength.

**Supplementary Table 1. Descriptive statistics and distribution characteristics of the composite scores.**

| **Variable** | **N** | **M** | **SD** | **Median** | **IQR** | **Skewness** | **Kurtosis** | **Shapiro-Wilk W** | **Shapiro_p** |
| --- | --- | --- | --- | --- | --- | --- | --- | --- | --- |
| POS (Total Score) | 439 | 32.71 | 5.41 | 32 | 9 | -0.335 | -0.486 | 0.946 | <0.001 |
| RES (Total Score) | 439 | 30.92 | 6.21 | 30 | 9 | -0.266 | 0.403 | 0.934 | <0.001 |
| TI (Total Score) | 439 | 9.89 | 3.59 | 9 | 5 | 0.927 | 0.529 | 0.901 | <0.001 |

N = 439. POS, Perceived Organisational Support; RES, resilience; TI, Turnover intention; SD, Standard Deviation; IQR, Interquartile Range. Normality was assessed using Shapiro–Wilk tests and visual diagnostics. Given deviations from normality and the ordinal nature of item responses, non-parametric analyses (Spearman correlations) were used. All variables represent total scores calculated by summing respective items.

**Supplementary Table 2. Spearman correlations among key study variables.**

| Variable | POS | RES | TI | Gender | Years of work experience | Job satisfaction |
| --- | --- | --- | --- | --- | --- | --- |
| POS | - | - | - | - | - | - |
| RES | 0.32*** | - | - | - | - | - |
| TI | -0.33*** | -0.36*** | - | - | - | - |
| Gender | -0.08 | 0.05 | -0.01 | - | - | - |
| Years of work experience | 0.02 | 0.07 | -0.14** | -0.21*** | - | - |
| Job satisfaction | 0.18*** | 0.34*** | -0.22*** | -0.01 | 0.03 | - |

POS, Perceived Organisational Support; RES, resilience; TI, Turnover intention. *p < 0.05, **p < 0.01, ***p < 0.001.

**Supplementary Table 3. Standardized path coefficients and indirect effects for the mediation models.**

| **Path** | **Model 0 (Baseline Model)** |  | **Model 1 (Covariate-Adjusted Model)** |  |
| --- | --- | --- | --- | --- |
|  | ***β* [95% CI]** | ***p*** | ***β* [95% CI]** | ***p*** |
| **Direct Effects** |  |  |  |  |
| POS→RES(a) | 0.317 [0.225, 0.409] | < 0.001 | 0.272 [0.159, 0.385] | < 0.001 |
| RES→TI(b) | -0.331[−0.437, −0.227] | < 0.001 | -0.329[-0.398, -0.260] | < 0.001 |
| POS→TI(c') | -0.170 [−0.262, −0.078] | 0.004 | -0.119 [-0.241, 0.003] | 0.056 |
| **Mediation Estimates** |  |  |  |  |
| Indirect effect (a×b) | -0.104 [-0.152, -0.058] | 0.004 | -0.089[-0.136, -0.042] | 0.048 |
| Total effect(c) | -0.274 [-0.357, -0.193] | < 0.001 | -0.208[-0.291, -0.127] | < 0.001 |

Note. N = 439. POS, Perceived Organisational Support; RES, resilience; TI, Turnover intention; CI, confidence intervals. Values are standardized coefficients (β). 95% confidence intervals are bias-corrected and accelerated (BCa) bootstrap intervals based on 2,000 resamples. In Model 1, covariates included (1 = male, 2 = female), years of work experience, and job satisfaction. Indirect effects are computed as the product of standardized path coefficients (a×b); total effects are computed as c′ + (a×b).

**Supplementary Table 4. Edge weight matrix of the POS-TI network.**

|  | **POS1** | **POS2** | **POS3** | **POS4** | **POS5** | **POS6** | **POS7** | **POS8** | **TI1** | **TI2** | **TI3** | **TI4** | **TI5** | **TI6** |
| --- | --- | --- | --- | --- | --- | --- | --- | --- | --- | --- | --- | --- | --- | --- |
| **POS1** | 0 | -0.060047 | 0.302253 | 0.427523 | 0.09727 | 0.078933 | 0.025604 | 0.008233 | 0 | 0 | 0 | 0 | 0 | 0 |
| **POS2** | -0.060047 | 0 | 0 | 0 | 0.306536 | 0.005562 | 0.074155 | 0.200966 | 0 | 0 | 0 | 0 | -0.019767 | -0.029594 |
| **POS3** | 0.302253 | 0 | 0 | 0.414333 | 0.008136 | 0 | 0.22984 | 0 | 0 | -0.042866 | 0 | -0.018029 | -0.039163 | -0.030673 |
| **POS4** | 0.427523 | 0 | 0.414333 | 0 | 0 | 0.186758 | 0.056037 | 0 | 0.006479 | 0 | 0 | 0 | 0 | -0.014766 |
| **POS5** | 0.09727 | 0.306536 | 0.008136 | 0 | 0 | 0 | 0 | 0.615022 | 0 | -0.012923 | -0.040921 | -0.008939 | 0 | 0 |
| **POS6** | 0.078933 | 0.005562 | 0 | 0.186758 | 0 | 0 | 0.543079 | 0 | 0 | 0.005051 | 0 | -0.026215 | 0.012 | 0.027882 |
| **POS7** | 0.025604 | 0.074155 | 0.22984 | 0.056037 | 0 | 0.543079 | 0 | 0 | 0 | -0.038817 | -0.05946 | -0.006276 | 0.095131 | 0 |
| **POS8** | 0.008233 | 0.200966 | 0 | 0 | 0.615022 | 0 | 0 | 0 | 0 | -0.051765 | 0 | 0 | 0.045444 | -0.030781 |
| **TI1** | 0 | 0 | 0 | 0.006479 | 0 | 0 | 0 | 0 | 0 | 0.580347 | 0.12994 | 0.264845 | 0 | 0.098853 |
| **TI2** | 0 | 0 | -0.042866 | 0 | -0.012923 | 0.005051 | -0.038817 | -0.051765 | 0.580347 | 0 | 0.341908 | 0 | 0 | 0 |
| **TI3** | 0 | 0 | 0 | 0 | -0.040921 | 0 | -0.05946 | 0 | 0.12994 | 0.341908 | 0 | 0.308488 | 0 | 0.036958 |
| **TI4** | 0 | 0 | -0.018029 | 0 | -0.008939 | -0.026215 | -0.006276 | 0 | 0.264845 | 0 | 0.308488 | 0 | 0.222854 | 0.183286 |
| **TI5** | 0 | -0.019767 | -0.039163 | 0 | 0 | 0.012 | 0.095131 | 0.045444 | 0 | 0 | 0 | 0.222854 | 0 | 0.444662 |
| **TI6** | 0 | -0.029594 | -0.030673 | -0.014766 | 0 | 0.027882 | 0 | -0.030781 | 0.098853 | 0 | 0.036958 | 0.183286 | 0.444662 | 0 |

POS, Perceived Organisational Support; TI, Turnover intention.

**Supplementary Table 5. Edge weight matrix of the POS-RES-TI network.**

|  | **POS1** | **POS2** | **POS3** | **POS4** | **POS5** | **POS6** | **POS7** | **POS8** | **RES1** | **RES10** | **RES2** | **RES3** | **RES4** | **RES5** | **RES6** | **RES7** | **RES8** | **RES9** | **TI1** | **TI2** | **TI3** | **TI4** | **TI5** | **TI6** |
| --- | --- | --- | --- | --- | --- | --- | --- | --- | --- | --- | --- | --- | --- | --- | --- | --- | --- | --- | --- | --- | --- | --- | --- | --- |
| **POS1** | 0 | -0.011975 | 0.294597 | 0.406028 | 0.097276 | 0.072232 | 0.01155 | 0 | 0.075461 | 0 | 0 | 0 | 0 | 0 | 0.005554 | 0 | 0 | 0 | 0 | 0 | 0 | 0 | 0 | 0 |
| **POS2** | -0.011975 | 0 | 0 | 0 | 0.288013 | 0.014607 | 0.069581 | 0.200611 | 0 | 0 | -0.025549 | -0.06248 | 0.053909 | 0 | -0.040898 | 0 | 0 | 0 | 0 | 0 | 0 | 0 | 0 | -0.039107 |
| **POS3** | 0.294597 | 0 | 0 | 0.406125 | 0.025395 | 0 | 0.225568 | 0 | 0.020291 | 0 | 0 | 0 | 0 | 0 | 0 | 0 | -0.020313 | 0 | 0 | -0.042179 | 0 | 0 | -0.023982 | -0.039972 |
| **POS4** | 0.406028 | 0 | 0.406125 | 0 | 0.000308 | 0.173738 | 0.049664 | 0 | 0 | 0 | 0 | 0 | 0 | 0 | 0.068257 | 0 | 0 | 0 | 0 | 0 | 0 | 0 | 0 | 0 |
| **POS5** | 0.097276 | 0.288013 | 0.025395 | 0.000308 | 0 | 0 | 0 | 0.604475 | 0.001299 | 0 | 0 | 0 | 0.003674 | 0 | -0.058288 | 0 | 0 | 0 | 0 | -0.014184 | -0.045535 | -0.018692 | 0 | 0 |
| **POS6** | 0.072232 | 0.014607 | 0 | 0.173738 | 0 | 0 | 0.506456 | 0 | 0.014903 | 0 | 0.015443 | 0.011257 | 0 | 0.048268 | 0 | 0 | 0 | 0 | 0 | 0 | 0 | 0 | 0.01245 | 0 |
| **POS7** | 0.01155 | 0.069581 | 0.225568 | 0.049664 | 0 | 0.506456 | 0 | 0.010332 | 0 | 0 | 0 | 0 | 0.12007 | 0 | 0 | 0 | 0 | 0 | 0 | -0.02592 | -0.045942 | 0 | 0.068558 | 0 |
| **POS8** | 0 | 0.200611 | 0 | 0 | 0.604475 | 0 | 0.010332 | 0 | 0 | 0 | 0 | 0 | 0 | 0 | 0 | 0 | 0 | 0.007966 | 0 | -0.049419 | 0 | 0 | 0.018117 | -0.01362 |
| **RES1** | 0.075461 | 0 | 0.020291 | 0 | 0.001299 | 0.014903 | 0 | 0 | 0 | 0.053448 | 0.319245 | 0.113685 | 0.186862 | 0.087133 | 0 | 0.088599 | 0 | 0.048615 | 0 | -0.026258 | 0 | 0 | 0 | 0 |
| **RES10** | 0 | 0 | 0 | 0 | 0 | 0 | 0 | 0 | 0.053448 | 0 | 0 | 0.04799 | 0.117736 | 0.050106 | 0.081575 | 0 | 0.214397 | 0.271613 | 0 | 0 | 0 | 0 | -0.005884 | 0 |
| **RES2** | 0 | -0.025549 | 0 | 0 | 0 | 0.015443 | 0 | 0 | 0.319245 | 0 | 0 | 0.350114 | 0.185643 | 0 | 0.129779 | 0 | 0 | 0 | 0.043878 | 0 | -0.021 | 0 | 0 | 0 |
| **RES3** | 0 | -0.06248 | 0 | 0 | 0 | 0.011257 | 0 | 0 | 0.113685 | 0.04799 | 0.350114 | 0 | 0 | 0.023578 | 0.060445 | 0.087144 | 0.182068 | 0 | 0 | 0 | 0 | 0 | 0 | 0 |
| **RES4** | 0 | 0.053909 | 0 | 0 | 0.003674 | 0 | 0.12007 | 0 | 0.186862 | 0.117736 | 0.185643 | 0 | 0 | 0.085362 | 0.001015 | 0.060762 | 0 | 0.18433 | -0.017851 | -0.036196 | 0 | 0 | 0 | 0.034135 |
| **RES5** | 0 | 0 | 0 | 0 | 0 | 0.048268 | 0 | 0 | 0.087133 | 0.050106 | 0 | 0.023578 | 0.085362 | 0 | 0.294357 | 0.148719 | 0.027132 | 0.203879 | 0 | 0 | 0 | -0.018228 | 0 | 0 |
| **RES6** | 0.005554 | -0.040898 | 0 | 0.068257 | -0.058288 | 0 | 0 | 0 | 0 | 0.081575 | 0.129779 | 0.060445 | 0.001015 | 0.294357 | 0 | 0.314198 | 0.05058 | 0 | 0 | -0.002553 | 0 | 0 | 0 | 0 |
| **RES7** | 0 | 0 | 0 | 0 | 0 | 0 | 0 | 0 | 0.088599 | 0 | 0 | 0.087144 | 0.060762 | 0.148719 | 0.314198 | 0 | 0.181941 | 0.138906 | -0.042119 | 0 | 0 | -0.05211 | 0.112286 | 0 |
| **RES8** | 0 | 0 | -0.020313 | 0 | 0 | 0 | 0 | 0 | 0 | 0.214397 | 0 | 0.182068 | 0 | 0.027132 | 0.05058 | 0.181941 | 0 | 0.272331 | 0 | 0.081467 | 0 | -0.00353 | -0.109657 | 0 |
| **RES9** | 0 | 0 | 0 | 0 | 0 | 0 | 0 | 0.007966 | 0.048615 | 0.271613 | 0 | 0 | 0.18433 | 0.203879 | 0 | 0.138906 | 0.272331 | 0 | 0 | 0 | 0.01308 | -0.041711 | 0 | 0 |
| **TI1** | 0 | 0 | 0 | 0 | 0 | 0 | 0 | 0 | 0 | 0 | 0.043878 | 0 | -0.017851 | 0 | 0 | -0.042119 | 0 | 0 | 0 | 0.563262 | 0.13168 | 0.255568 | 0 | 0.103548 |
| **TI2** | 0 | 0 | -0.042179 | 0 | -0.014184 | 0 | -0.02592 | -0.049419 | -0.026258 | 0 | 0 | 0 | -0.036196 | 0 | -0.002553 | 0 | 0.081467 | 0 | 0.563262 | 0 | 0.344048 | 0 | 0.011492 | 0 |
| **TI3** | 0 | 0 | 0 | 0 | -0.045535 | 0 | -0.045942 | 0 | 0 | 0 | -0.021 | 0 | 0 | 0 | 0 | 0 | 0 | 0.01308 | 0.13168 | 0.344048 | 0 | 0.301126 | 0 | 0.040398 |
| **TI4** | 0 | 0 | 0 | 0 | -0.018692 | 0 | 0 | 0 | 0 | 0 | 0 | 0 | 0 | -0.018228 | 0 | -0.05211 | -0.00353 | -0.041711 | 0.255568 | 0 | 0.301126 | 0 | 0.221032 | 0.187014 |
| **TI5** | 0 | 0 | -0.023982 | 0 | 0 | 0.01245 | 0.068558 | 0.018117 | 0 | -0.005884 | 0 | 0 | 0 | 0 | 0 | 0.112286 | -0.109657 | 0 | 0 | 0.011492 | 0 | 0.221032 | 0 | 0.431868 |
| **TI6** | 0 | -0.039107 | -0.039972 | 0 | 0 | 0 | 0 | -0.01362 | 0 | 0 | 0 | 0 | 0.034135 | 0 | 0 | 0 | 0 | 0 | 0.103548 | 0 | 0.040398 | 0.187014 | 0.431868 | 0 |
|  |  |  |  |  |  |  |  |  |  |  |  |  |  |  |  |  |  |  |  |  |  |  |  |  |

POS, Perceived Organisational Support; RES, resilience; TI, Turnover intention.

**Supplementary Fig. 1: Histograms and Q–Q plots**

**a**, Histogram, density curve, Q–Q plot, and box plot for the total Perceived Organisational Support score; **b**, Histogram, density curve, Q–Q plot, and box plot for the total Resilience score; **c**, Histogram, density curve, Q–Q plot, and box plot for the total Turnover Intention score. Normality of the three scale totals was assessed using Shapiro–Wilk tests and visual diagnostics. POS, Perceived Organisational Support; RES, resilience; TI, Turnover Intention.

**a**


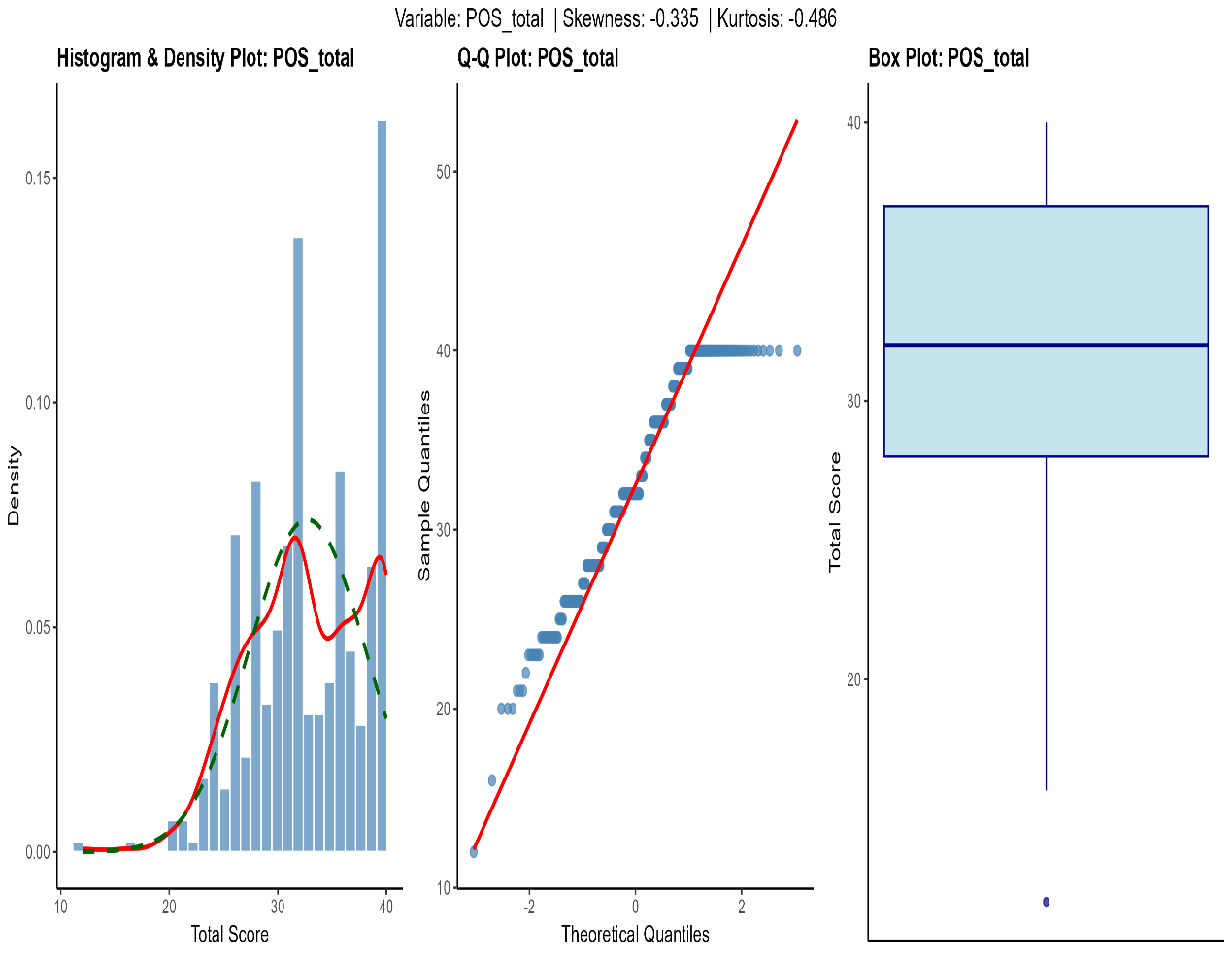


**b**


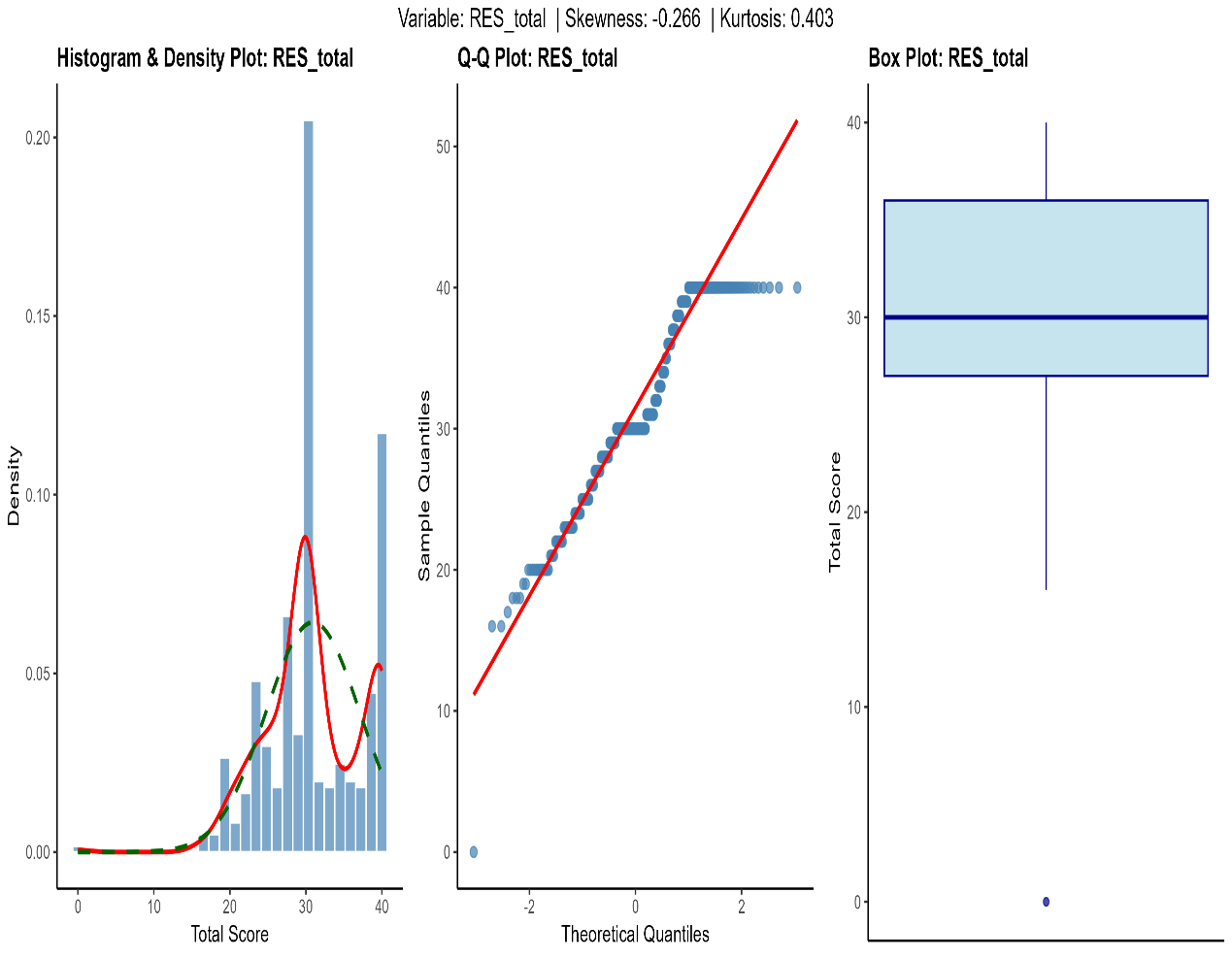


**c**


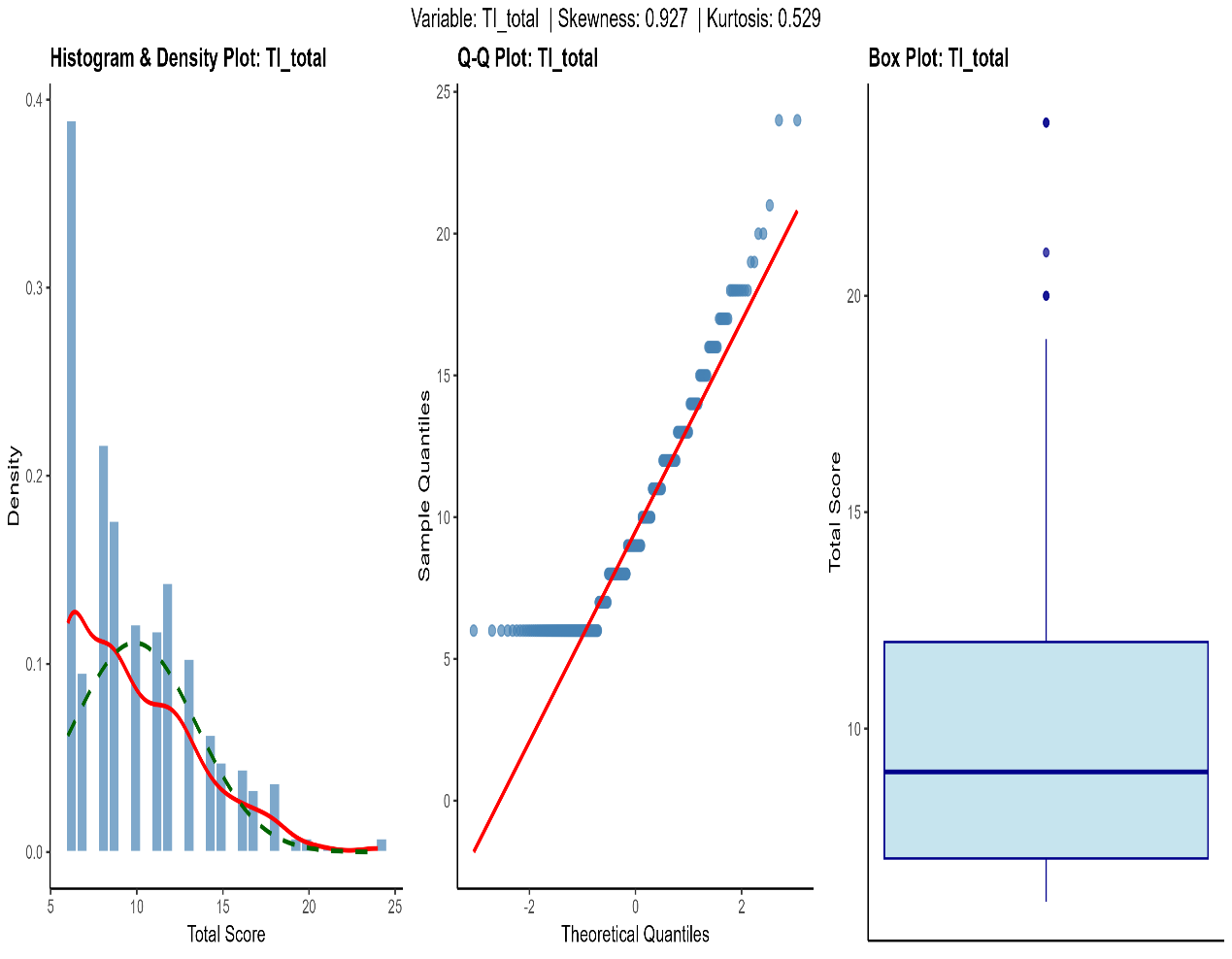


**Supplementary Fig. 2: Accuracy of edge weights and bridge strength.**

**a** and **b** are non-parametric bootstrap tests of edge weights and bridge strength, respectively, of the POS-TI network. **c** and **d** are non-parametric bootstrap tests of edge weights and bridge strength, respectively, of the POS-RES-TI network. The shades represent the 95% confidence interval for the indices.POS, Perceived Organisational Support; RES, resilience; TI, Turnover intention.

**a**

**
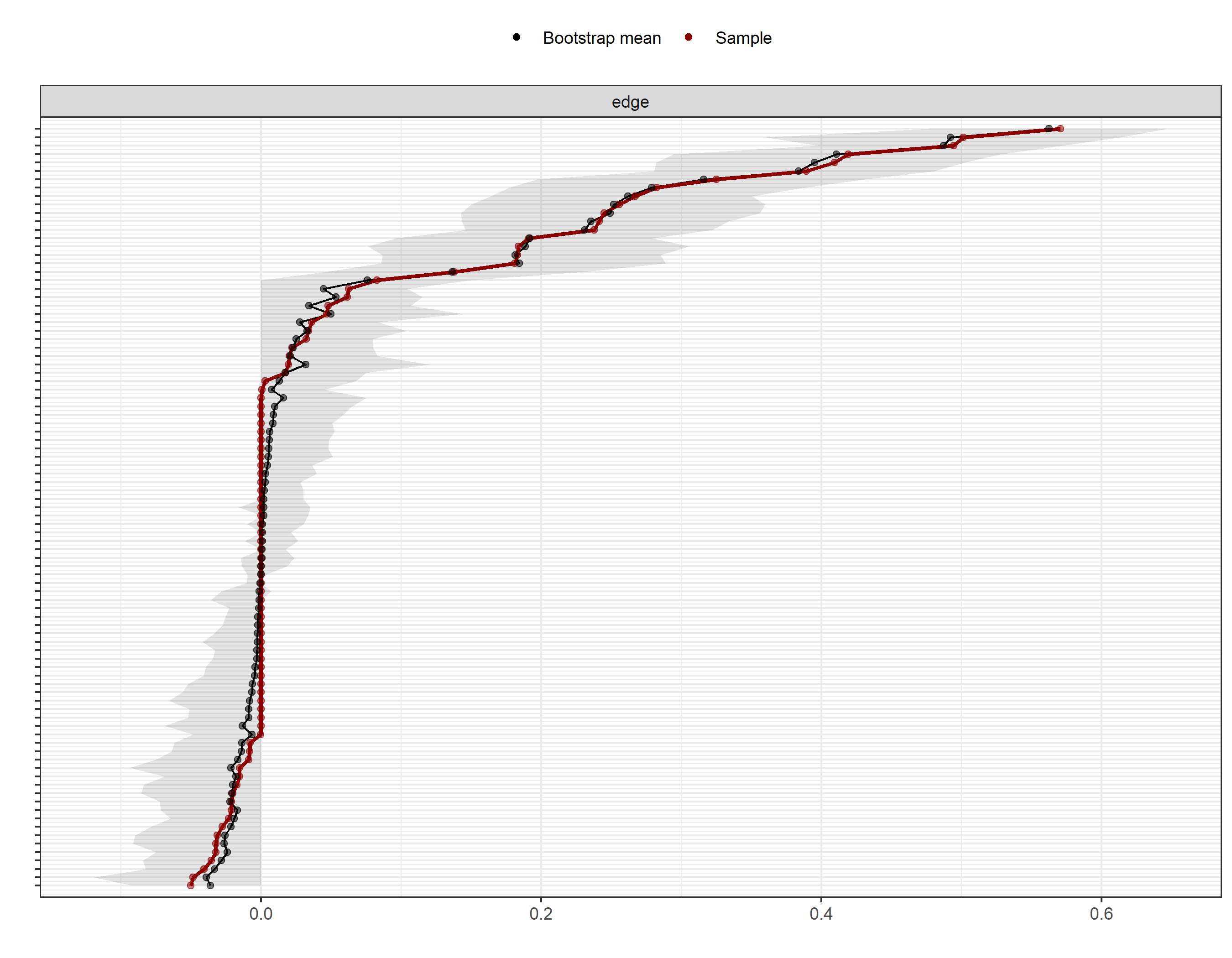
**

**b**

**
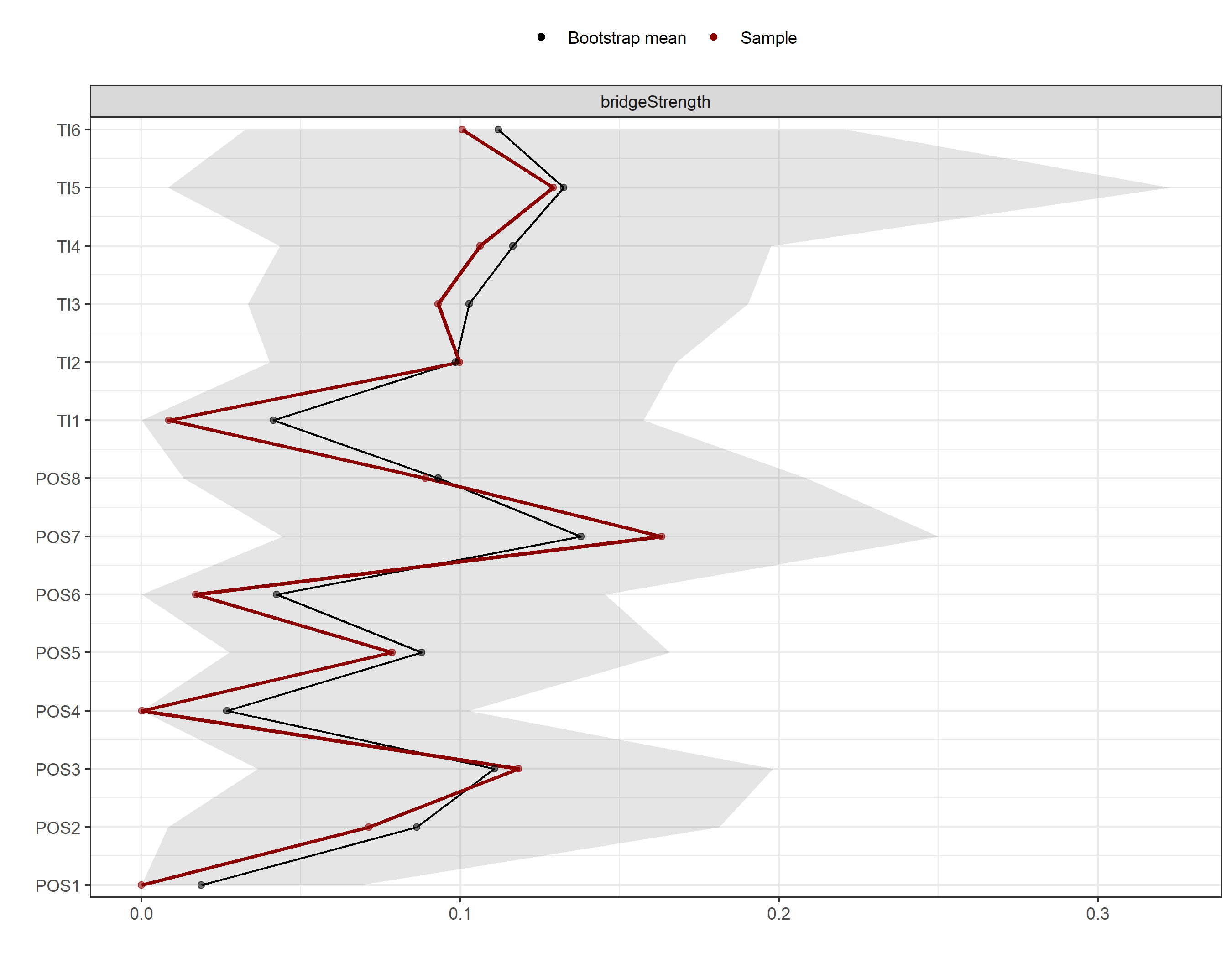
**

**c**

**
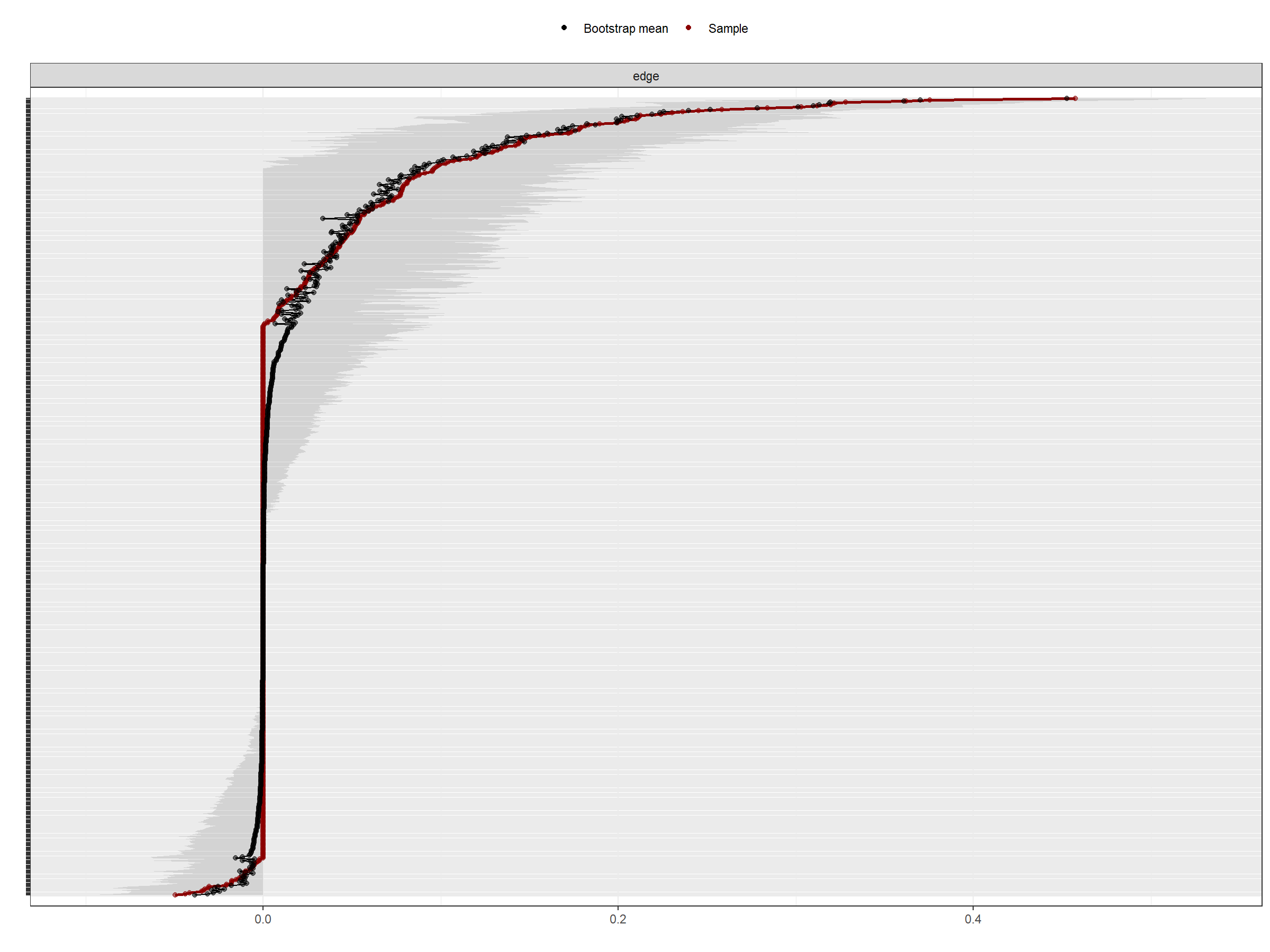
**

**d**

**
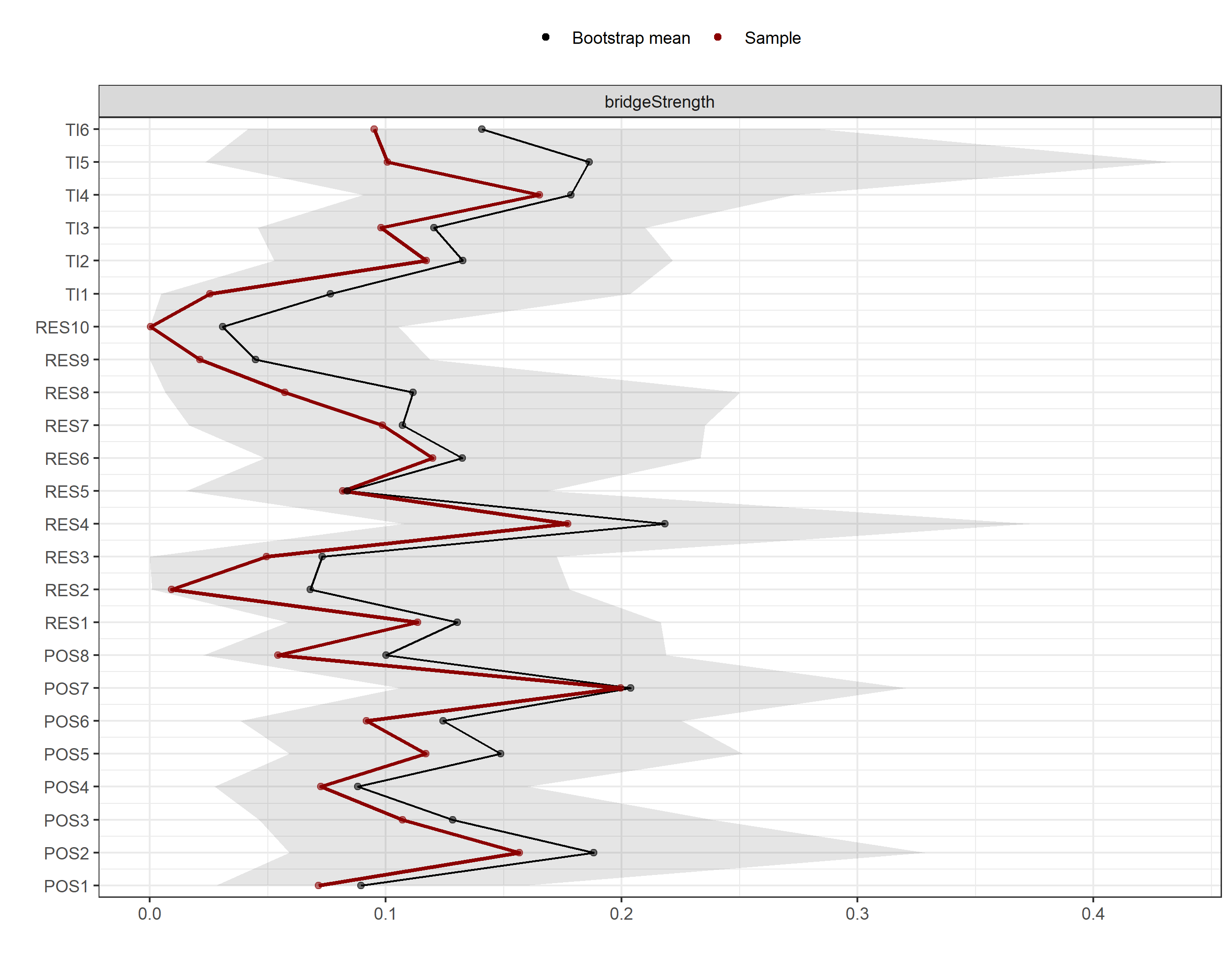
**

**Supplementary Fig. 3: Stability of node centrality indices and edges.**

**a** and **b** show the stability of node centrality indices (strength, betweenness, and closeness) and edge weights, respectively, for the POS-TI network. **c** and **d** show the stability of node centrality indices (strength, betweenness, and closeness) and edge weights, respectively, for the POS-RES-TI network. The shades represent the 95% confidence interval for the indices. POS, Perceived Organisational Support; RES, resilience; TI, Turnover intention.

**a**


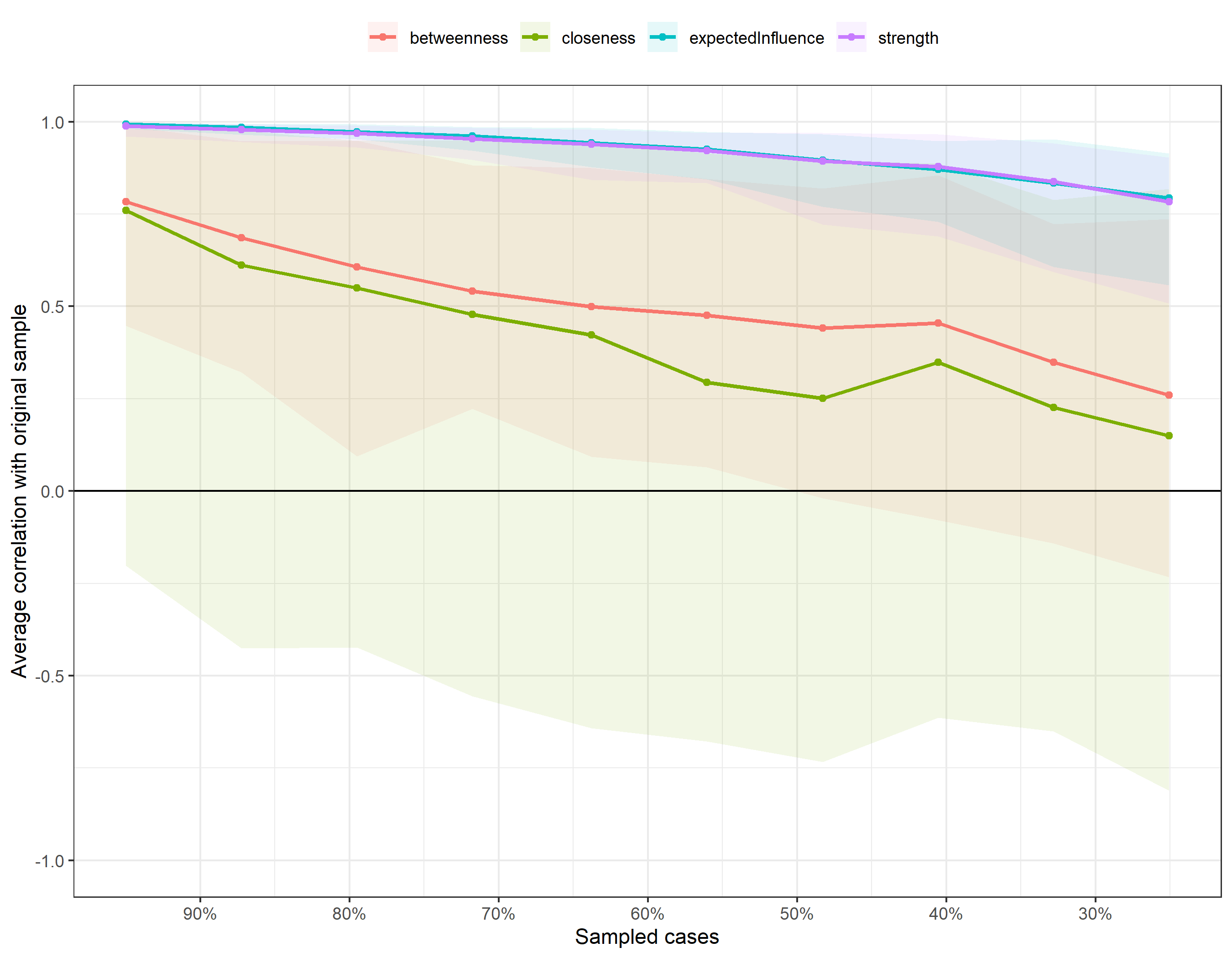


**b**

**
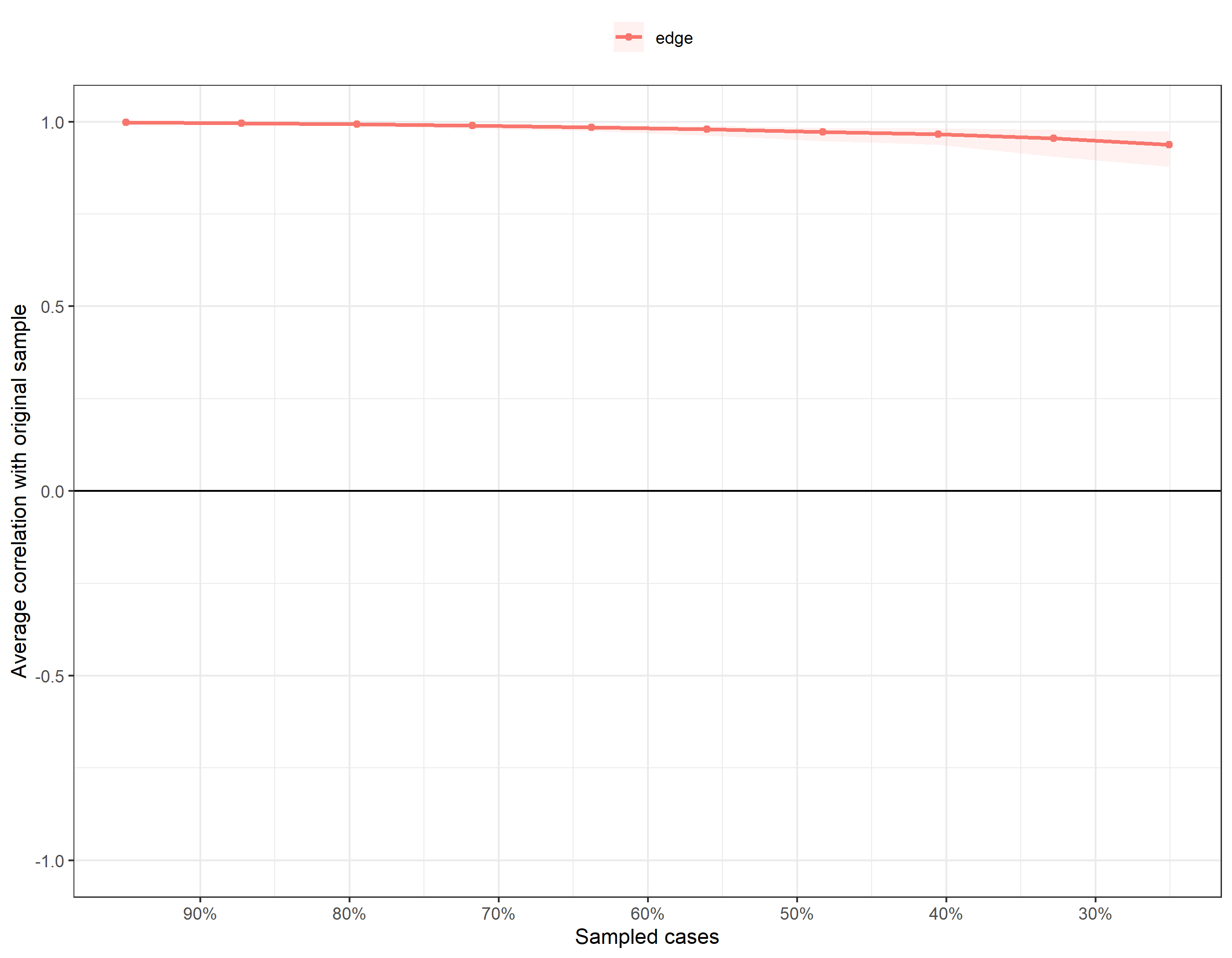
**

**c**

**
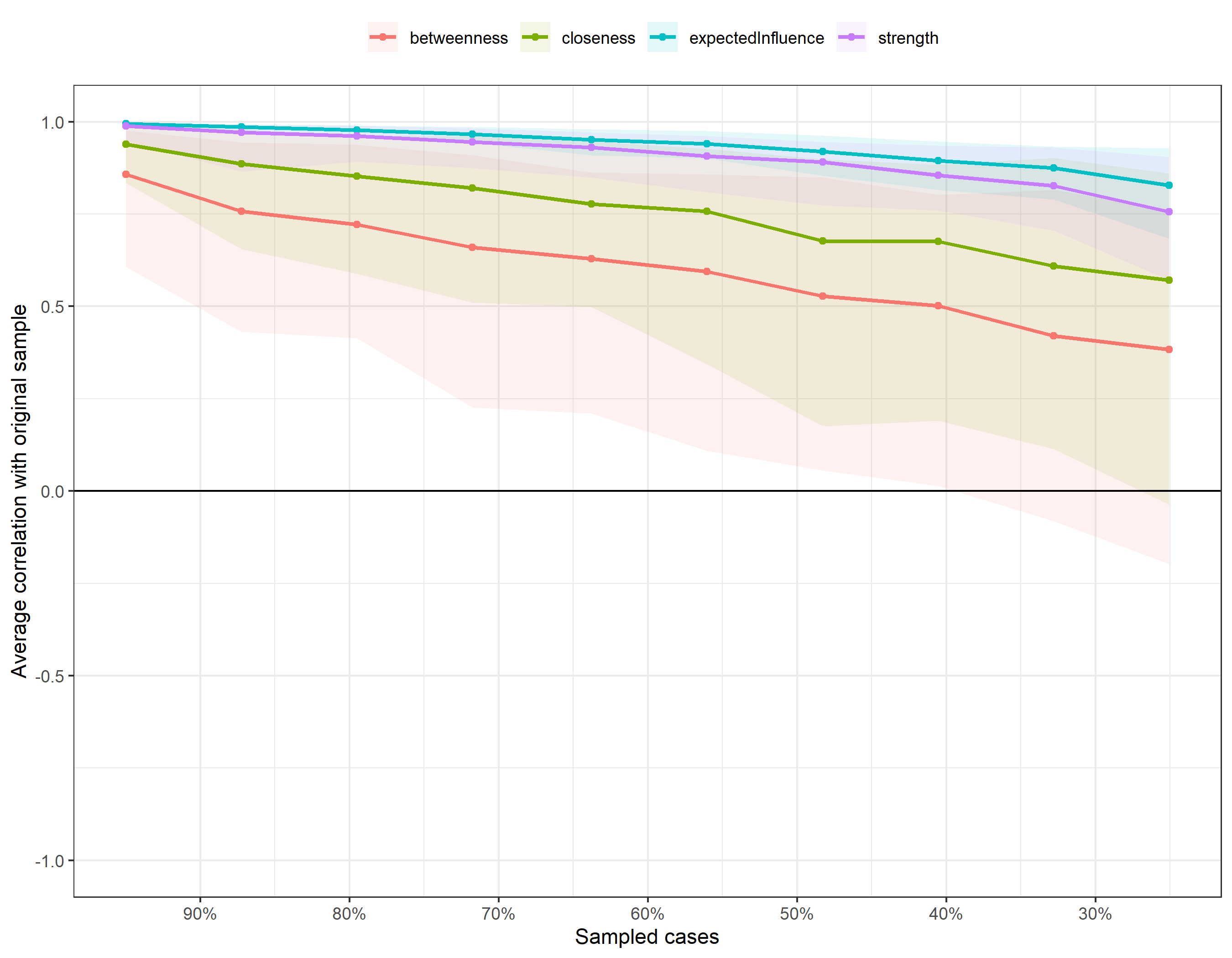
**

**d**

**
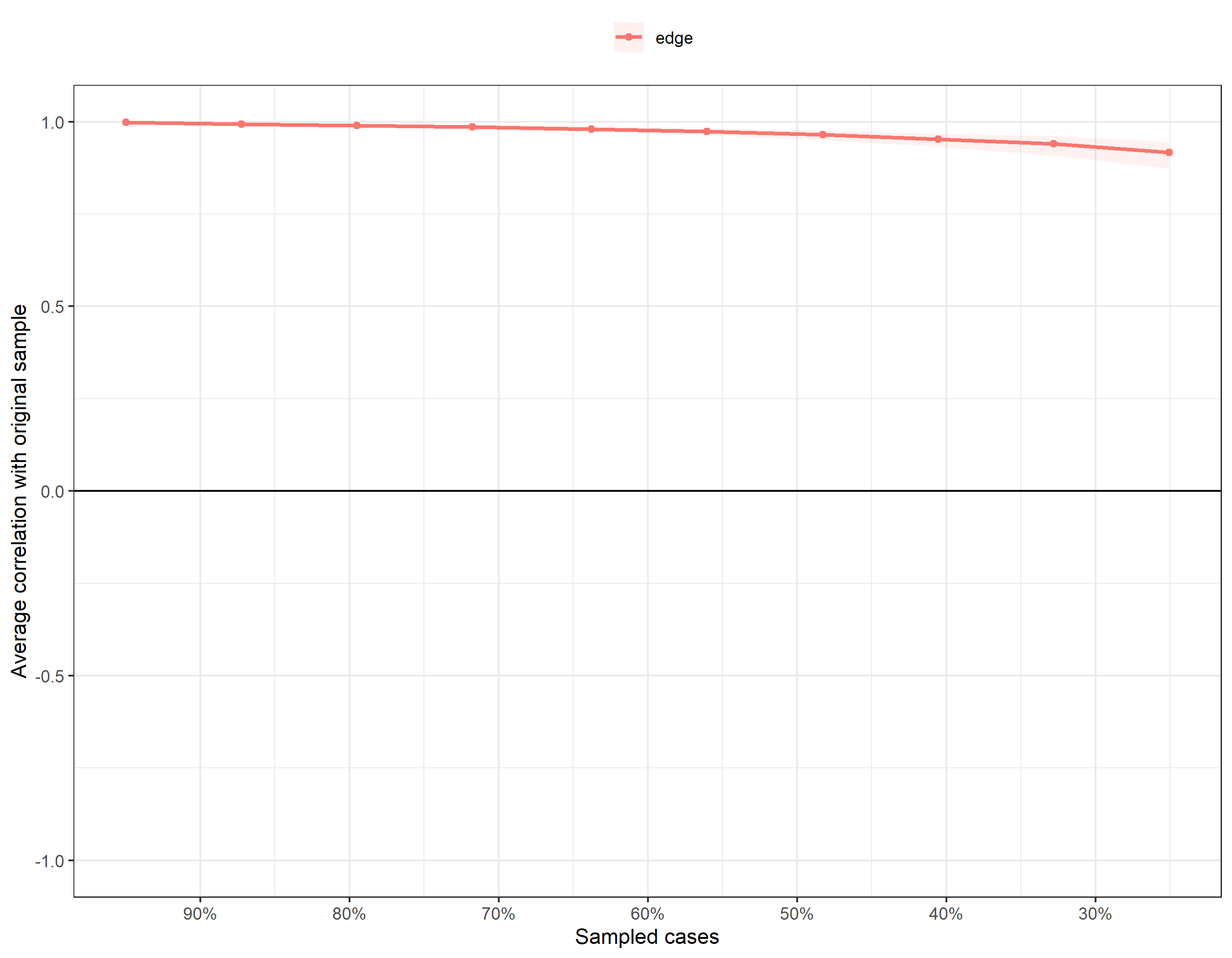
**

**Supplementary Fig. 4: Bootstrapped difference test of edge weights and expected influence. a** and **b** show the difference tests of edge weights and expected influence, respectively, for the POS-TI network. **c** and **d** show the difference tests of edge weights and expected influence, respectively, for the POS-RES-TI network. Dark squares indicate that the edge weights or the expected influence of the corresponding nodes differ significantly from one another (a = 0.05). Grey squares mean that the edge weights or the expected influence of the corresponding nodes do not significantly differ from one another. In the plots of the edge weight difference test, blue boxes represent positive correlations while red boxes represent negative correlations. POS, Perceived Organisational Support; RES, resilience; TI, Turnover intention.

**a**

**
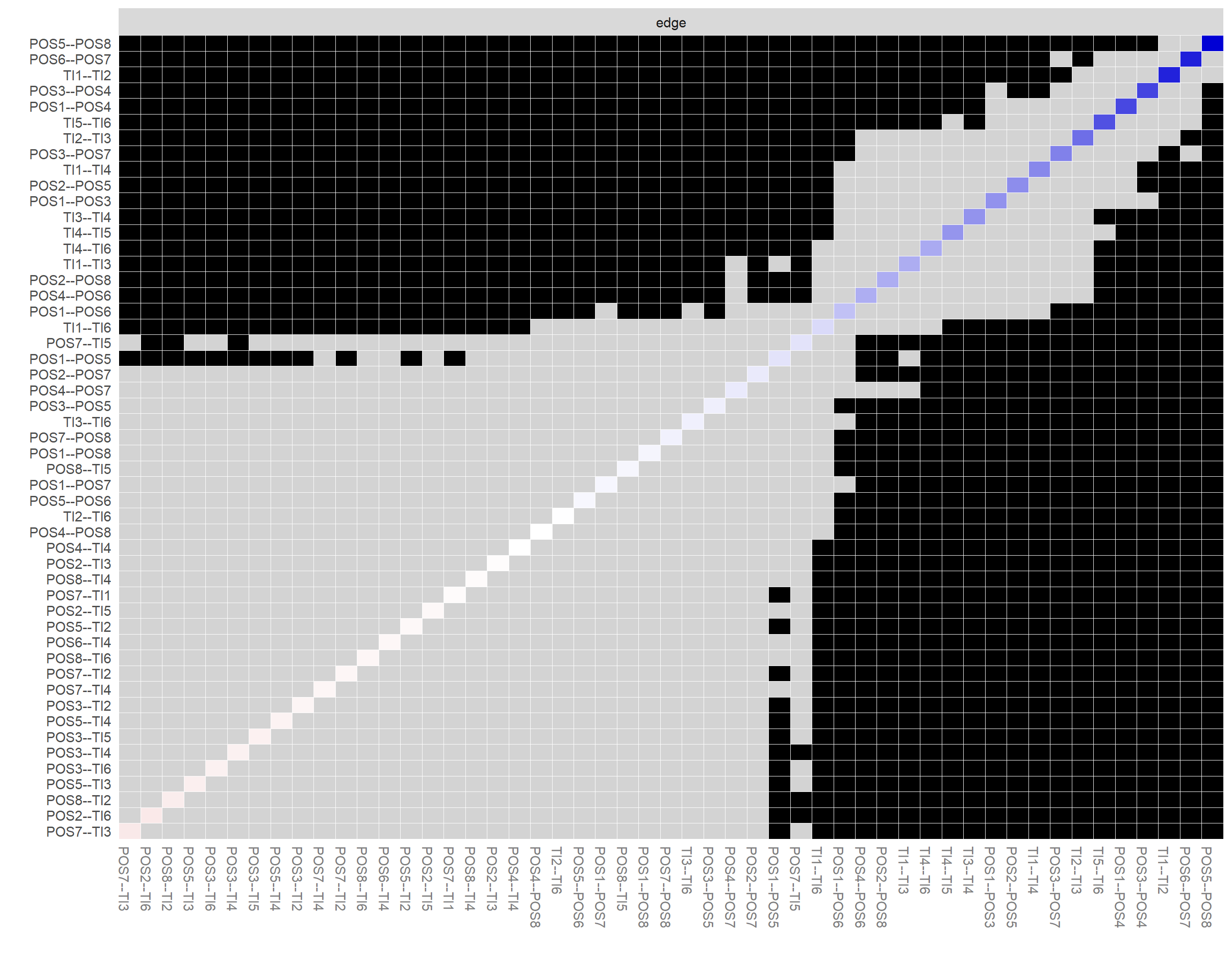
**

**b**

**
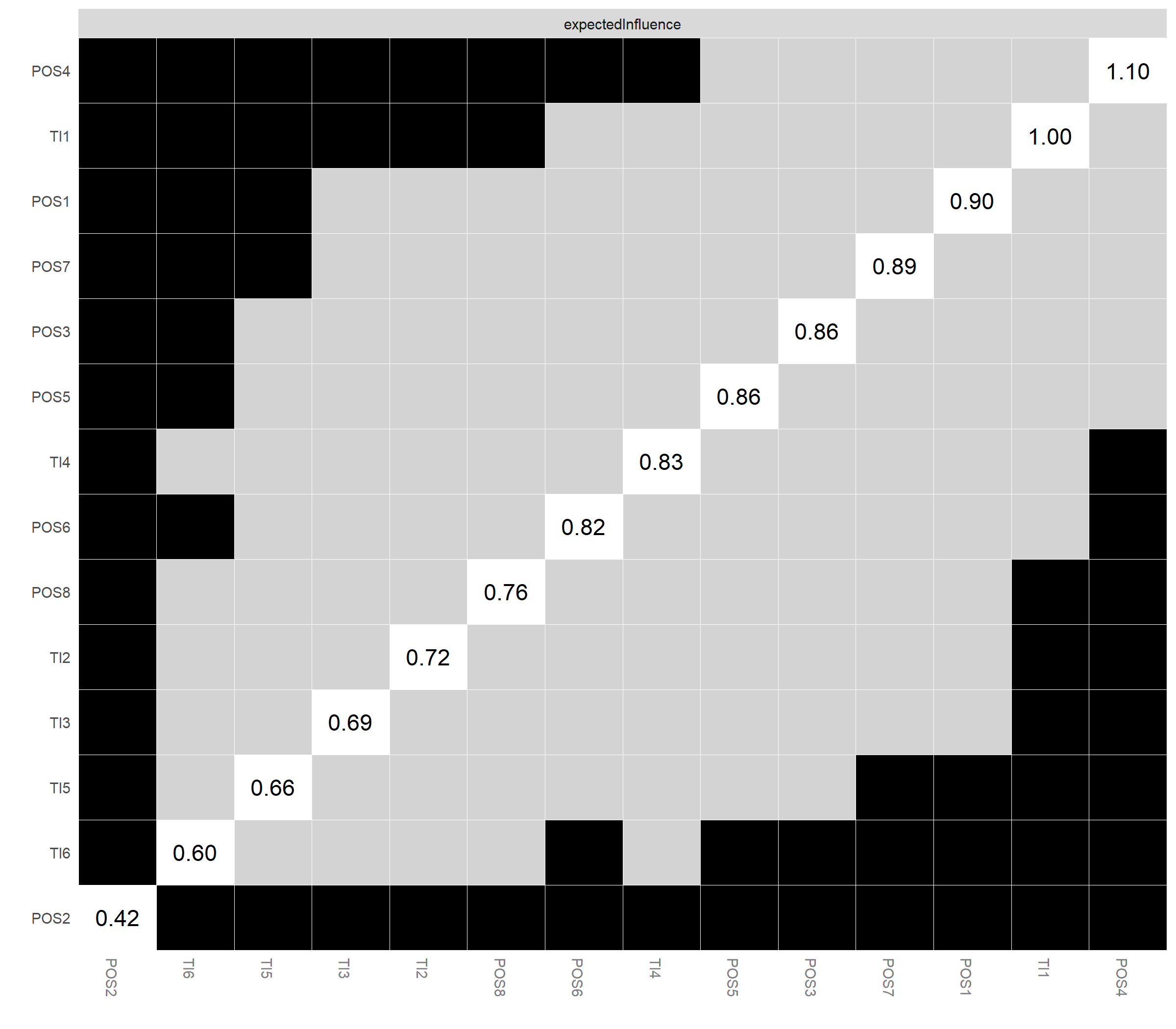
**

**c**

**
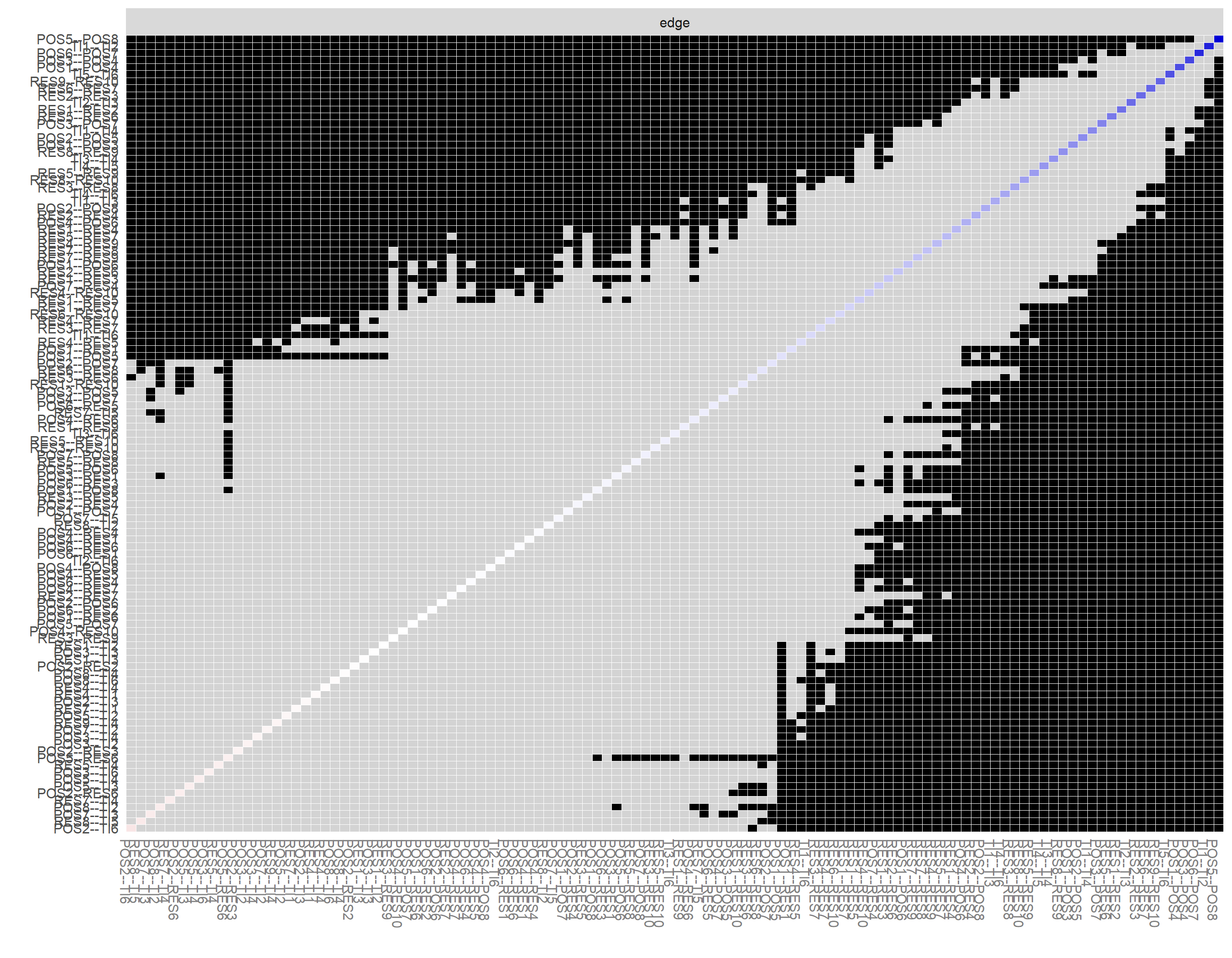
**

**d**

**
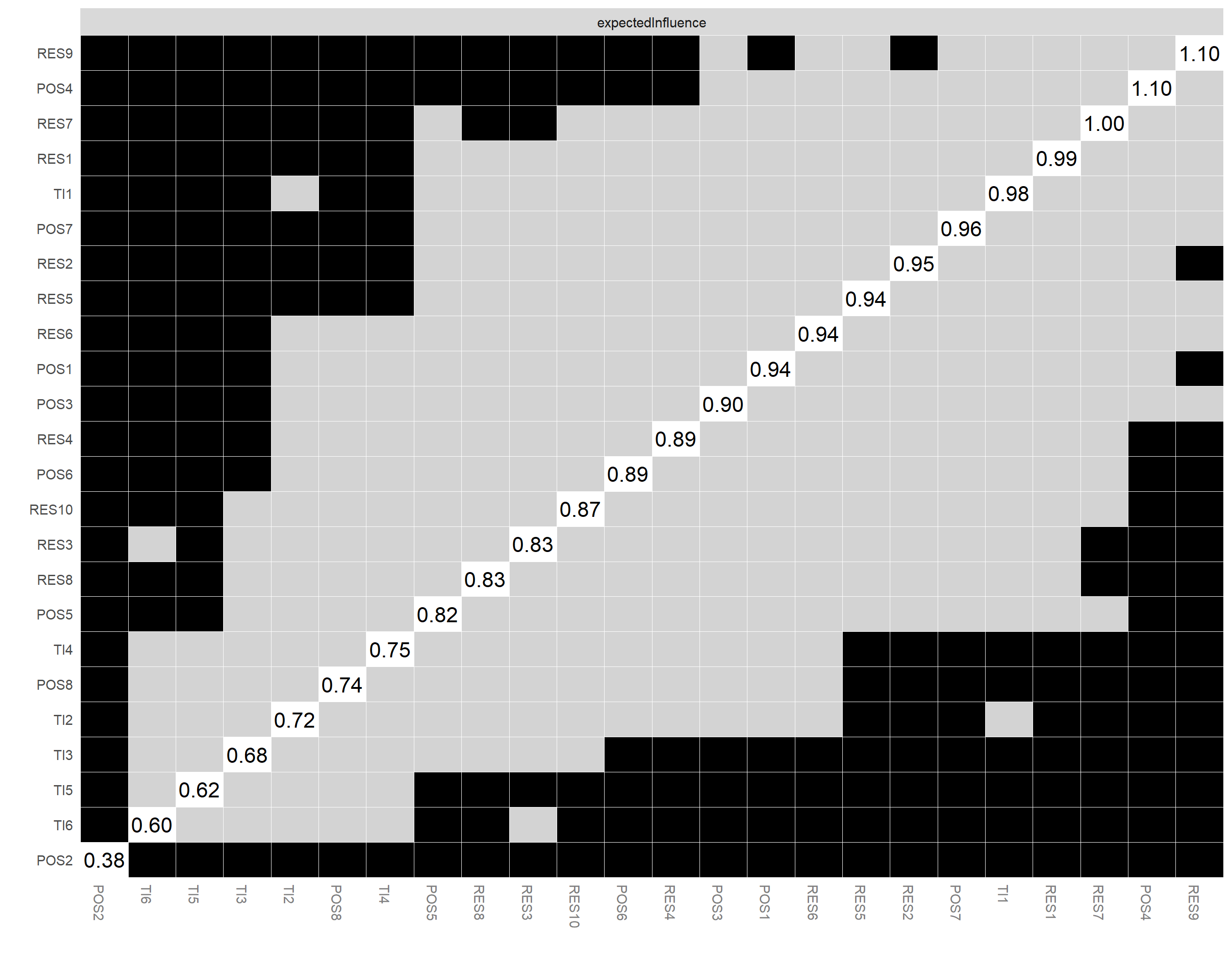
**

**Supplementary Fig. 5: Network Comparison tests of the** **POS-RES-TI networks of the two equally split subsamples.**

**a** illustrates the network structure with the bridge items of subsample 1, and **b** illustrates the network structure with the bridge nodes of subsample 2. **c** shows the bridge expected influence of subsample 1, and **d** shows the bridge expected influence of subsample 2. POS, Perceived Organisational Support; RES, resilience; TI, Turnover intention.

**a**

**
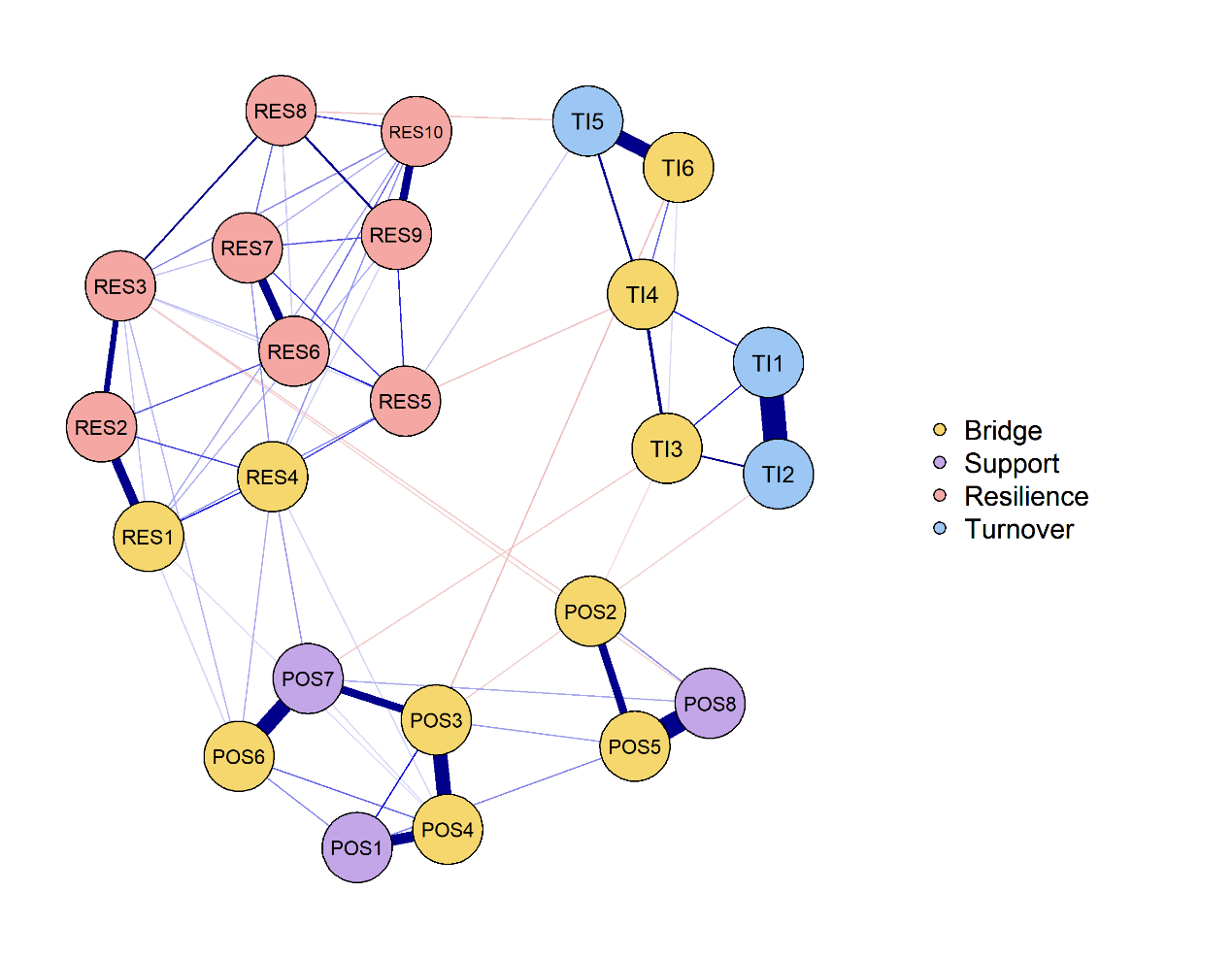
**

**b**

**
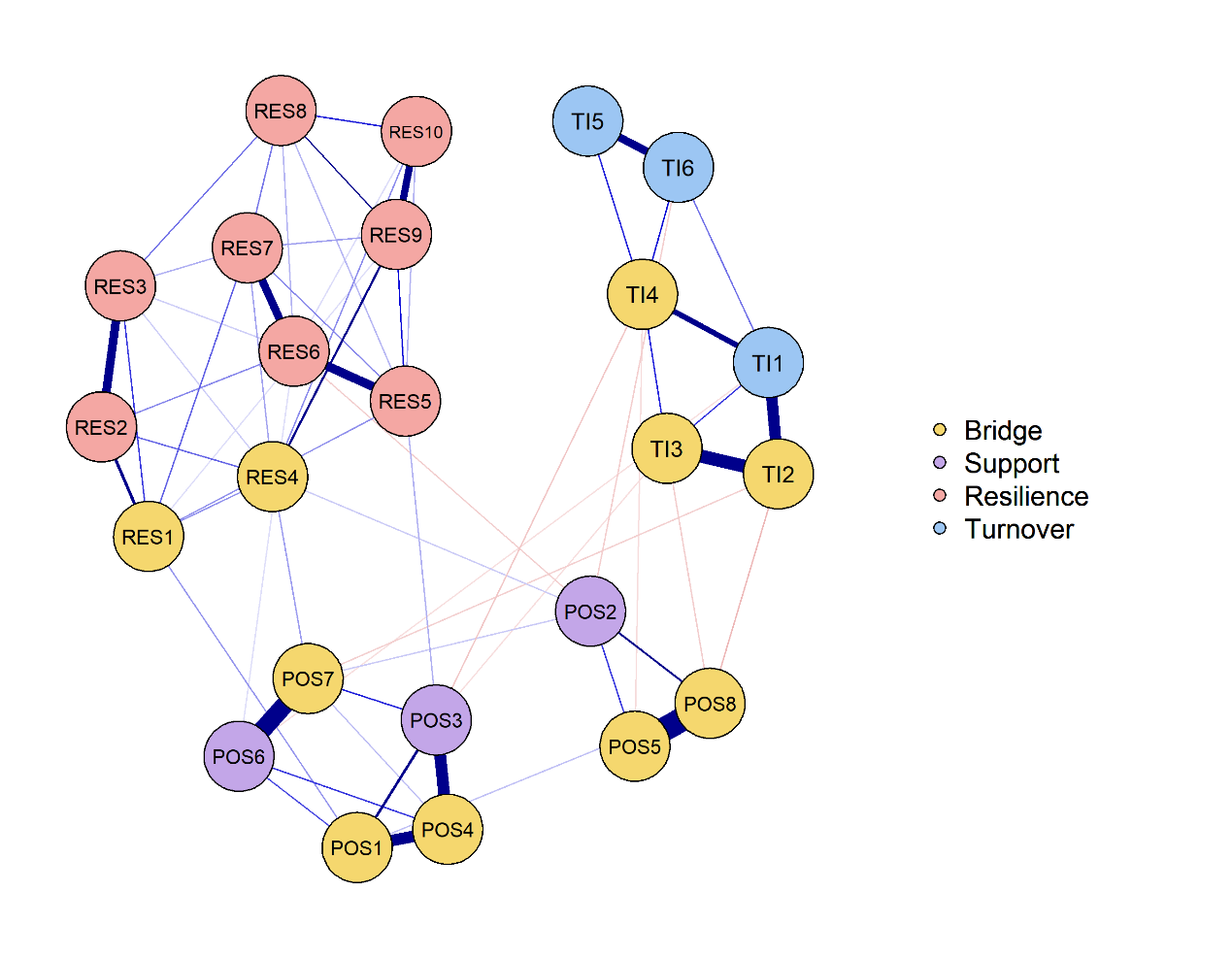
**

**c**

**
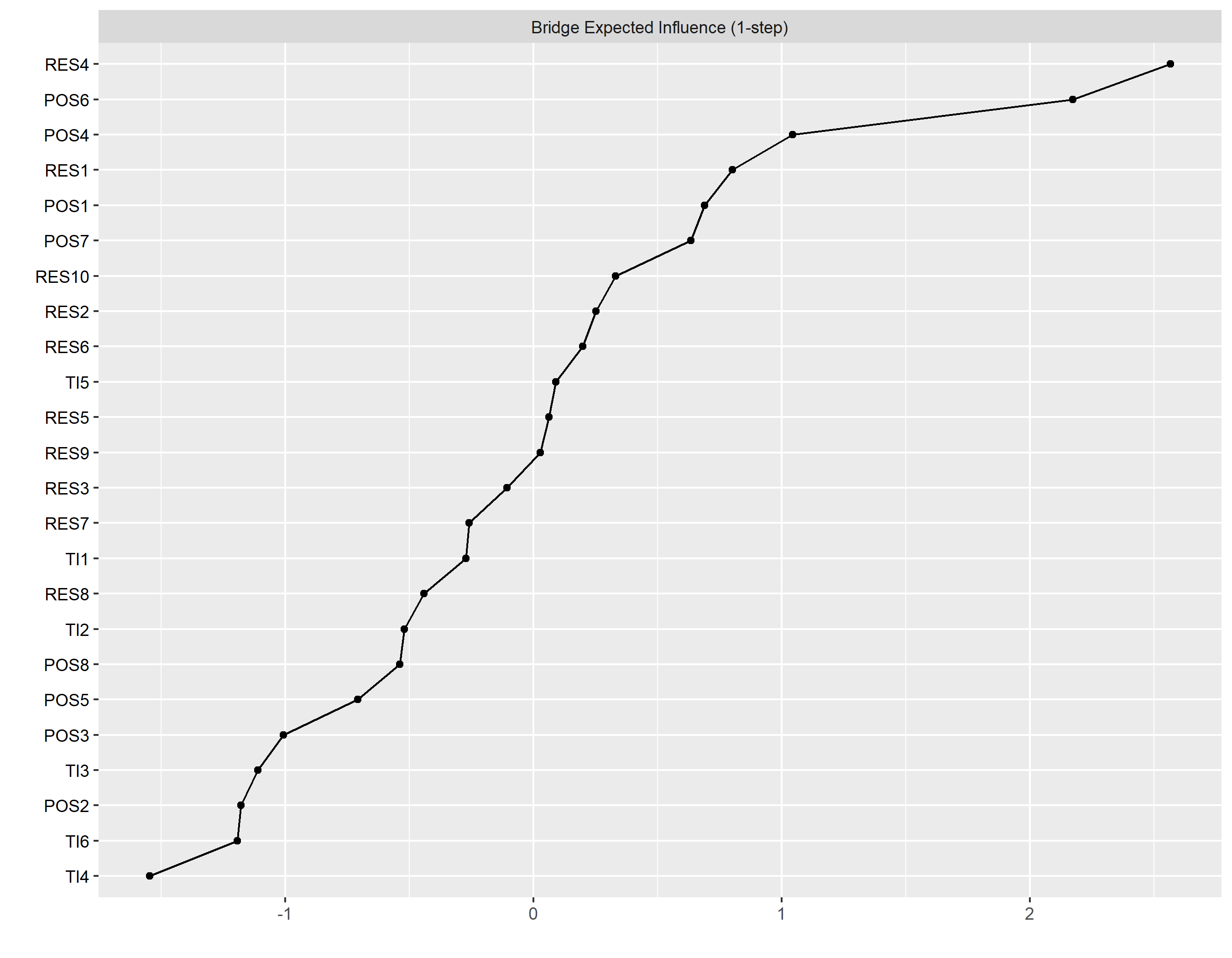
**

**d**

**
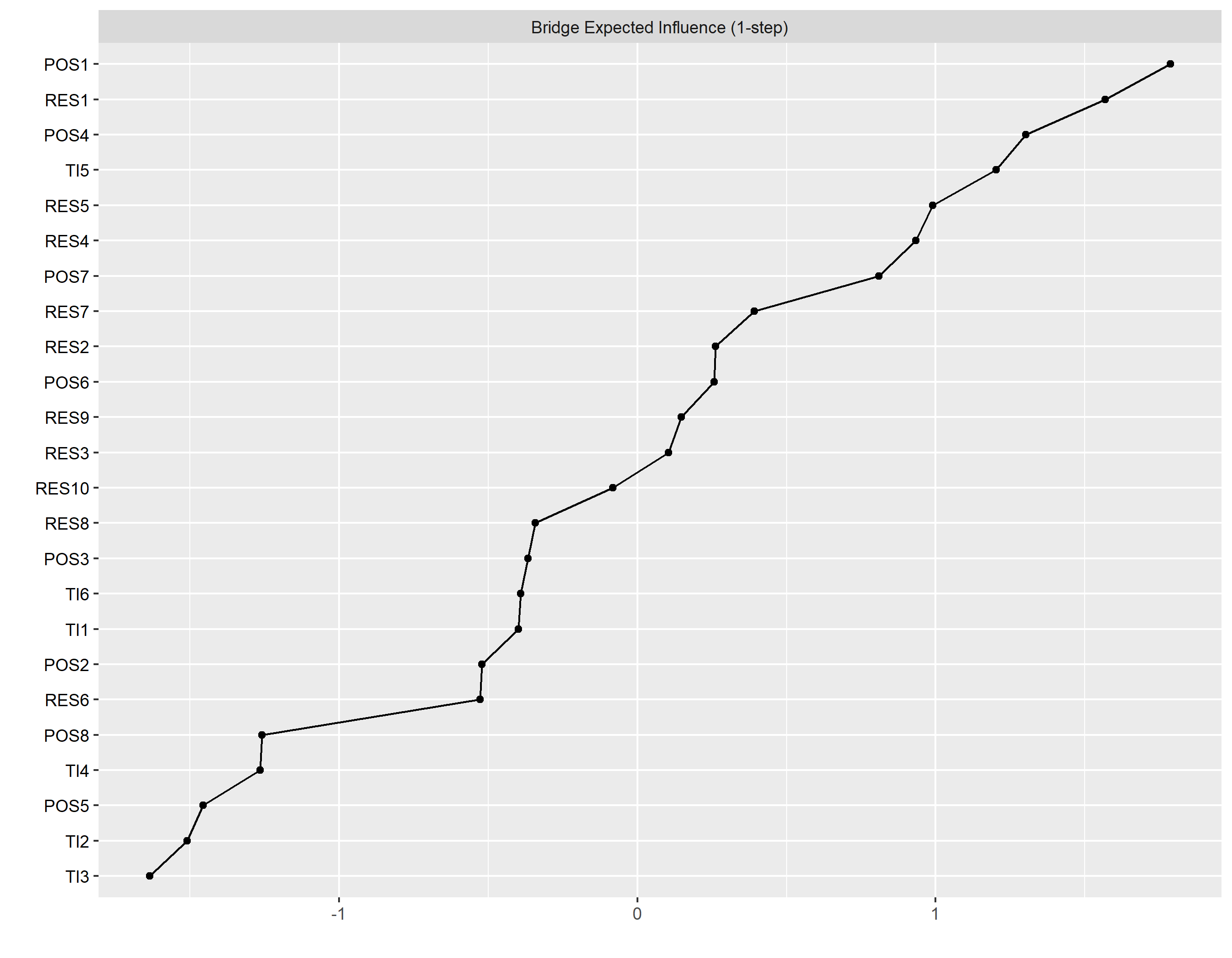
**

**Supplementary Fig. 6: Bootstrapped difference tests of bridge expected influence.**

**a** shows the bootstrapped difference test of the POS-TI network. **b** shows the bootstrapped difference test of the POS-RES-TI network. Dark squares indicate that the bridge expected influence of the corresponding nodes differ significantly from one another (a = 0.05). Grey squares mean that the bridge expected influence of the corresponding nodes do not significantly differ from one another. POS, Perceived Organisational Support; RES, resilience; TI, Turnover intention.

**a**

**
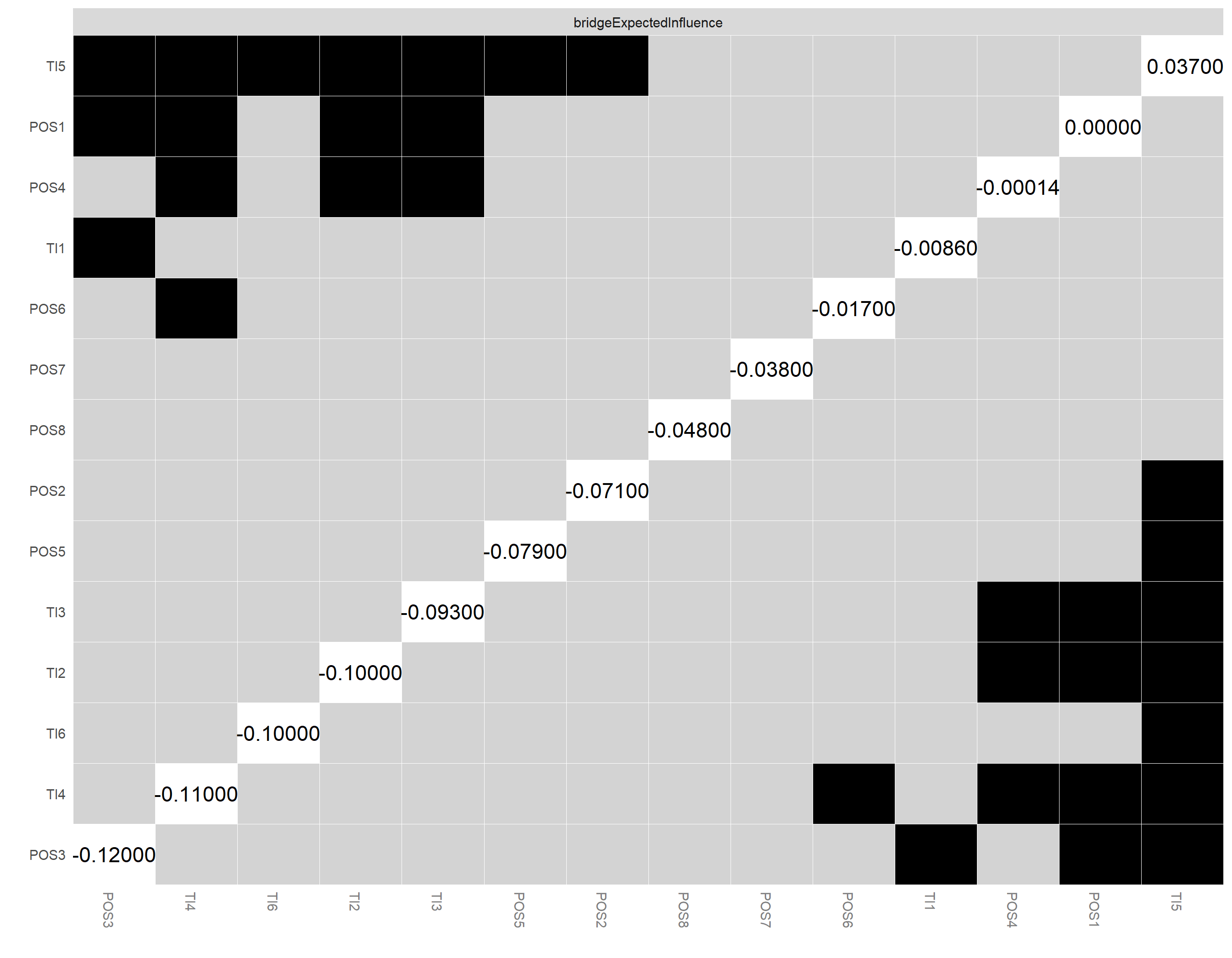
**

**b**

**
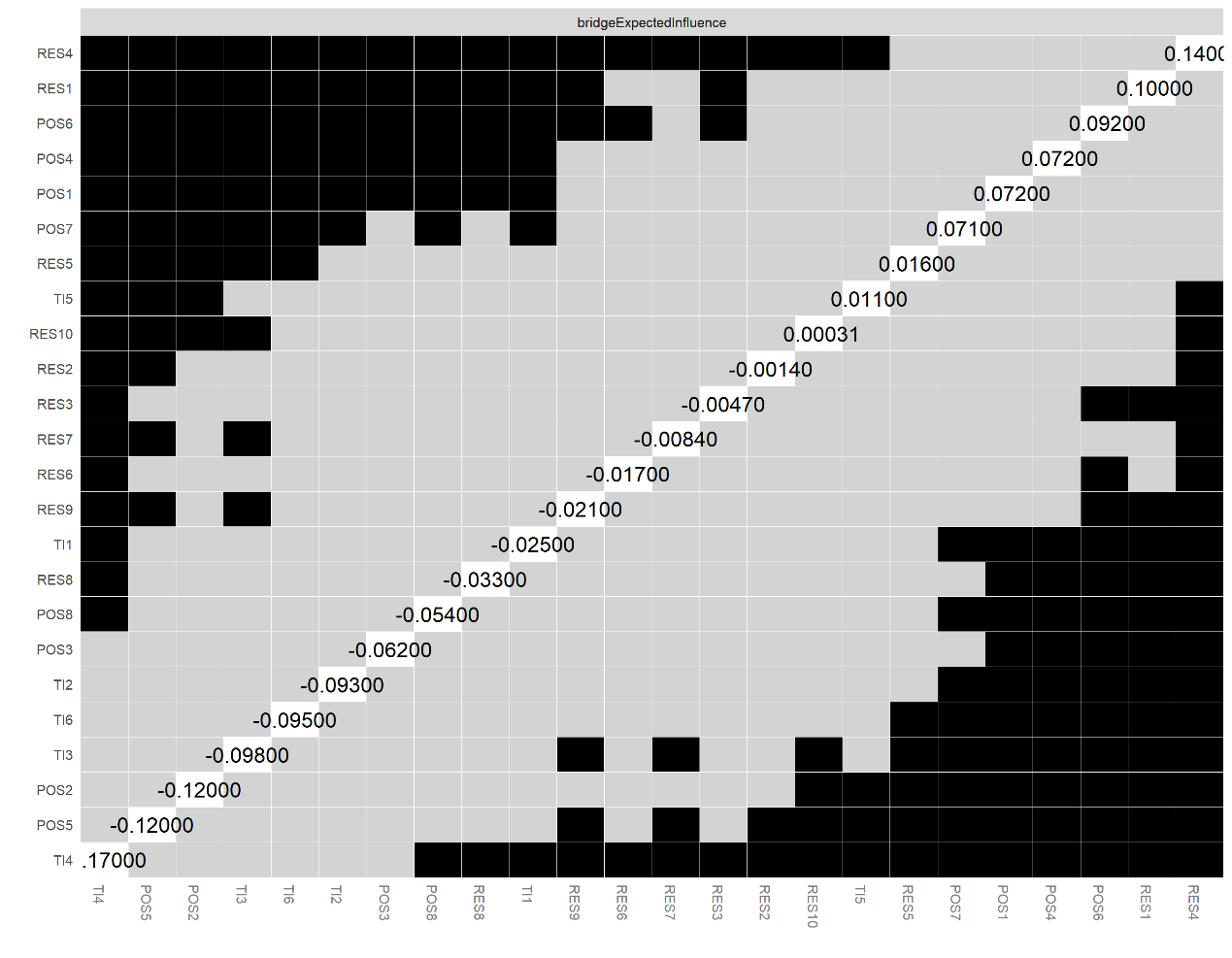
**

**Supplementary Fig. 7: Node centrality indices: strength, betweenness, closeness, and**

**expected influence.**

**a** shows the node centrality indices of the POS-TI network. **b** shows the node centrality indices of the POS-RES-TI network. The centrality indices include strength, betweenness, closeness, and expected influence, which reflect the importance of each node within the network in terms of connectivity, control of information flow, and potential influence over other nodes. Perceived Organisational Support; RES, resilience; TI, Turnover intention.

**a**

**
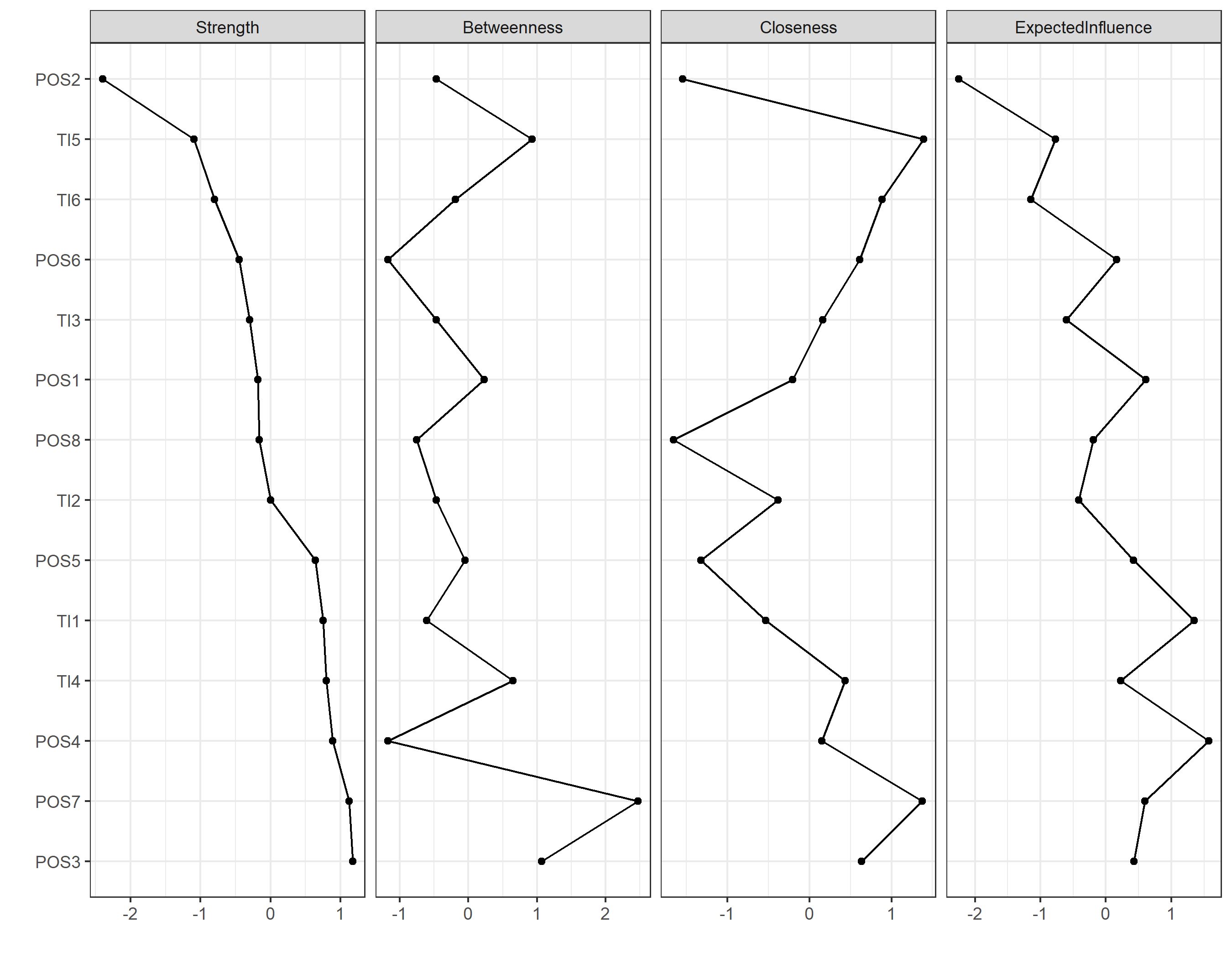
**

**b**

**
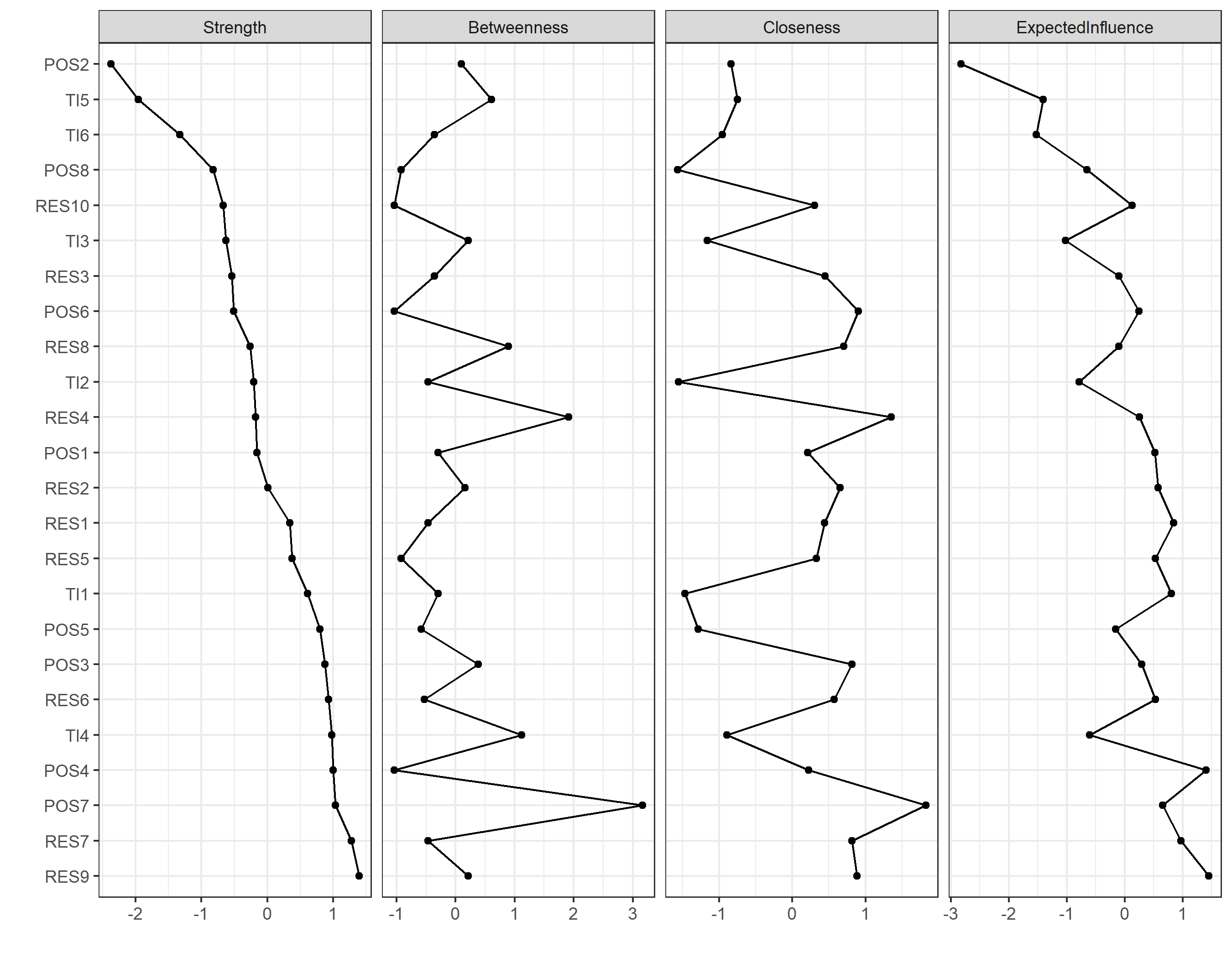
**

**Supplementary Fig. 8: Bridge strength.**

**a** illustrates the bridge strength of the POS-TI network and **b** illustrates the bridge strength of the POS-RES-TI network from the highest to the lowest. Bridge strength reflects the extent to which a node connects different bridge item communities, indicating its potential importance in inter-community interactions. Perceived Organisational Support; RES, resilience; TI, Turnover intention.

**a**

**
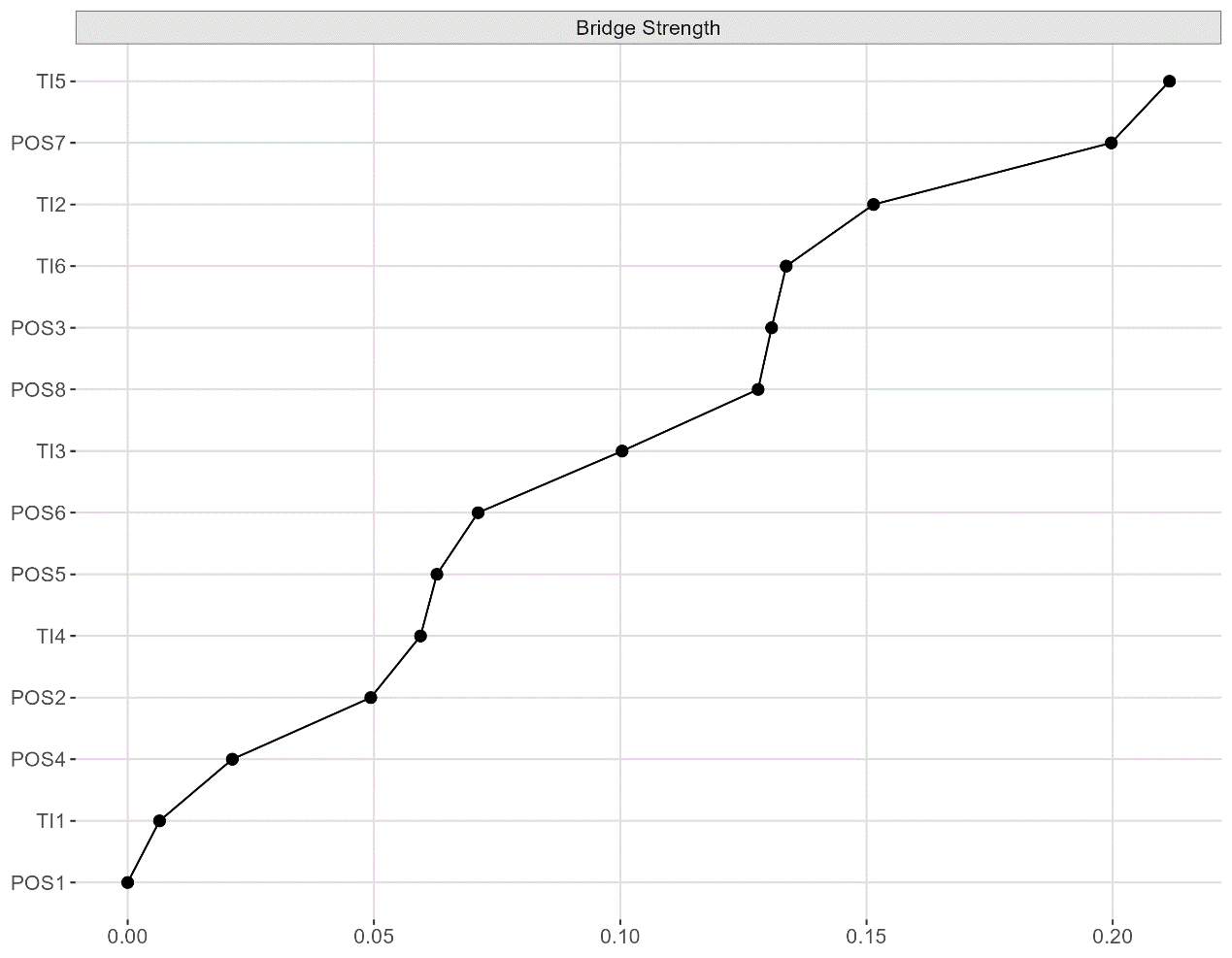
**

**b**

**
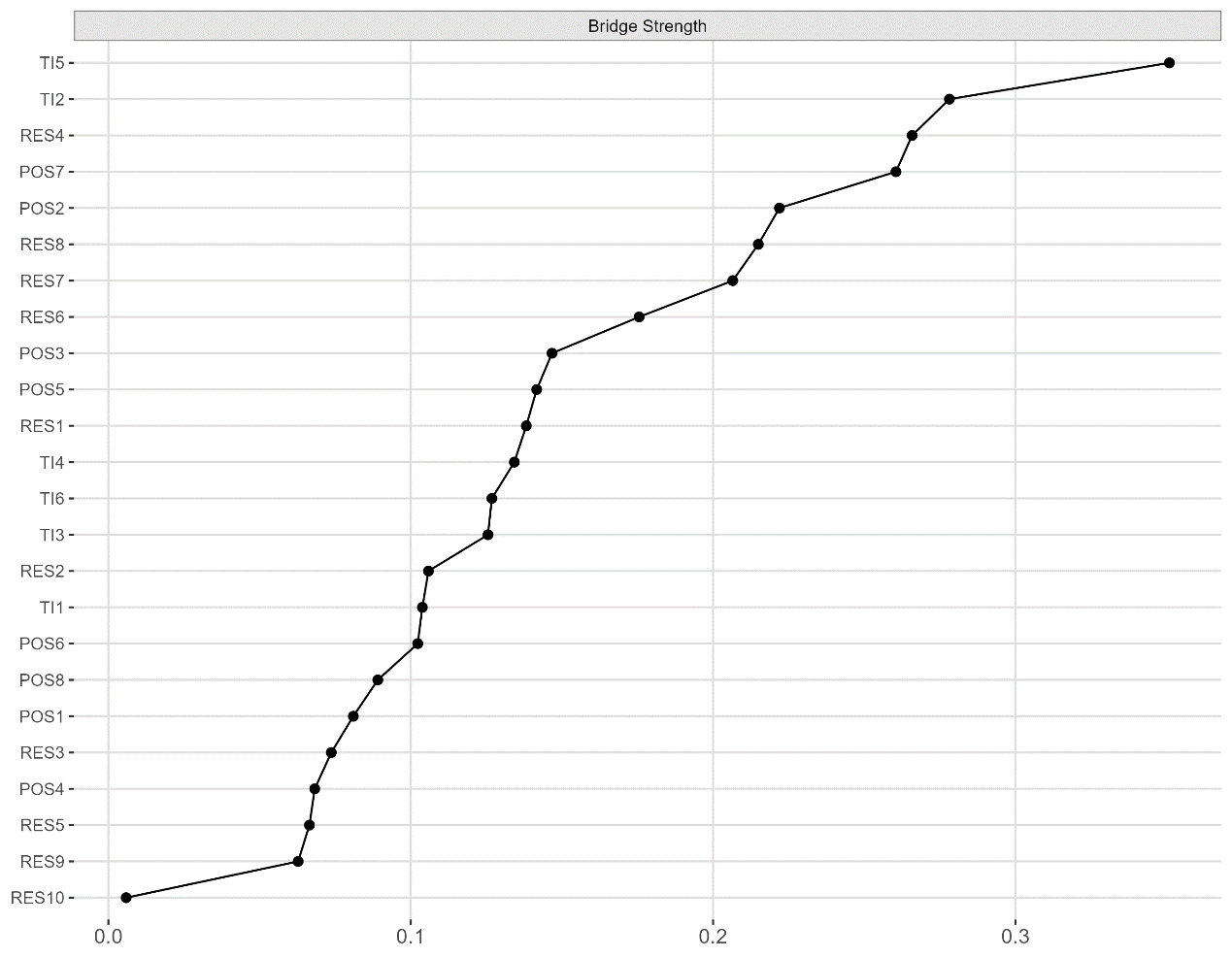
**
